# Mechanism and application of Taq DNA polymerase in TaqMan qPCR

**DOI:** 10.3389/fbioe.2026.1773703

**Published:** 2026-05-20

**Authors:** Qiuyu Cao, Ziling Zou, Duanru Cao, Xiaofei Zhang, Tao Shu, Zhipeng Qu, Yanchang Zhao

**Affiliations:** Life Sciences Division, Nanjing Vazyme Biotech Co., Ltd., Nanjing, Jiangsu, China

**Keywords:** nuclease activity, probe cleavage efficiency, probe melting curve, Taq pol, TaqMan qPCR

## Abstract

TaqMan quantitative real-time PCR (qPCR) is widely used in molecular biology due to its high specificity, sensitivity, and multiplexing capability. Taq DNA polymerase (Taq pol) plays a central role by simultaneously catalyzing DNA synthesis and cleaving fluorogenic probes, yet the mechanistic interplay between these activities remains incompletely characterized. We systematically quantified Taq pol nuclease and polymerase activities during extension and probe cleavage. A novel screening assay integrating both extension and cleavage readouts was developed to accurately evaluate Taq pol performance. Several engineered Taq variants and KlenTaq (lacking the 5′-3′ exonuclease domain) were subsequently analyzed. Our analysis revealed a synergistic relationship between polymerase extension and probe cleavage activities. Two Taq variants exhibited significantly enhanced probe cleavage efficiency, while KlenTaq demonstrated markedly stronger strand displacement activity. These findings elucidate the molecular mechanisms governing Taq pol activity during qPCR. The identified variants with improved cleavage efficiency are promising for multiplex detection, whereas KlenTaq’s enhanced strand displacement supports specialized applications such as probe melting curve analysis. This work provides both theoretical insights and practical tools to optimize qPCR assays and engineer next-generation polymerases.

## 1 Introduction

Taq DNA polymerase (Taq pol), originally isolated from the thermophilic bacterium *Thermus aquaticus* (Chien et al., 1976), was the first thermostable DNA polymerase utilized in PCR technology (Brock and Freeze, 1969). It exhibits a high amplification efficiency and excellent thermostability, and serves as the core enzyme in PCR reactions. Since Saiki first utilized this enzyme in PCR technology in 1988 (Mullis et al., 1986; Mullis and Faloona, 1987; Saiki et al., 1988), it has become one of the most fundamental and commonly used tools in molecular biology research. It is widely applied in fields such as gene cloning (Saiki et al., 1988), DNA sequencing (Innis et al., 1988; Chen, 2014), and molecular diagnostics (Fouchier et al., 2000; Khodakov et al., 2016), and has provided a critical basis for the development of various PCR techniques (Tozaki et al., 2021; Li et al., 2023; Li et al., 2025; Shahrajabian and Sun, 2024).

Taq pol is a DNA-dependent DNA polymerase that possesses 5′-3′ polymerase activity. It extends the 3′end of a primer to build a new complementary strand, and 5′-3′ exonuclease activity, which can degrade a DNA stand under specific conditions (Lyamichev et al., 1993; Kim et al., 1995). It is the synergistic actions of these two activities that enables effective quantification in fluorescent quantitative PCR (qPCR). In TaqMan qPCR, Taq pol not only catalyzes DNA synthesis, but also cleaves the labeled probe that is specifically bound to the target sequence to release a quantifiable fluorescent signal. Thus, the effectiveness of this detection mechanism is highly dependent on the 5′-3′ exonuclease activity (Holland et al., 1991; Huang et al., 2021). Therefore, an in-depth study of the mechanisms of Taq pol and an optimization of its functional properties not only contributes to the understanding of the molecular mechanisms of DNA polymerases, but also provides a theoretical basis and experimental foundation for the development and innovation of qPCR technology. This is critical for the development of more efficient and sensitive qPCR reagents and detection methods.

Common detection methods used in qPCR include SYBR Green I, a DNA binding-dye, and TaqMan, a probe-based method, with each having its own technical characteristics and application scenarios (Wilhelm and Pingoud, 2003). The SYBR Green I method detects PCR products by non-specifically binding double-stranded DNA. This offers the advantage of low cost and a simple operation, but provides poor specificity (Morrison et al., 1998). In contrast, the TaqMan probe method achieves high specificity by detecting target sequence-specific probes, and thus exhibits higher detection accuracy in complex samples (Schmittgen et al., 2000). While previous studies have reported that higher SYBR Green I concentrations can inhibit Taq pol amplification (Eischeid, 2011), the impact of probes on the amplification process remains incompletely understood. During extension, Taq pol displaces the 5′end of the probe, forming a “fork-like” structure, and initiates a hydrolysis reaction that cleaves each 5′end nucleotide sequentially, releasing the reporter from the quencher (Holland et al., 1991; Lyamichev et al., 1993). However, how probe cleavage may impact amplification efficiency remains unclear, and it is unclear if this process involves the cooperation of other activities, such as strand displacement activity (Barnes et al., 2021) or 5′-flap endonuclease activity (mediated by its 5′-3′ exonuclease activity) (Lyamichev et al., 1993). This process not being fully illuminated limits obtaining an in-depth understanding of the functional mechanisms of Taq pol, and affects the optimization of its application in qPCR and other detection technologies.

To further characterize the mechanism of Taq pol in probe cleavage, multiple reaction models were constructed to analyze its exonuclease and 5′-flap endonuclease activities. The results showed that the nuclease activity of Taq pol can function independently of its polymerase activity, which is consistent with previous reports (Lorenz, 1974). However, during TaqMan qPCR, probe cleavage is not entirely dependent on exonuclease activity but is instead the result of the synergistic actions of multiple enzymatic activities. To compare the performance of different Taq variants in TaqMan qPCR, four engineered variants developed through distinct strategies were selected for evaluation. Two Taq variants, S-Taq (Wang, 2004) and Taq-E507K (Arezi et al., 2014), were engineered to improve processivity and inhibitor tolerance through two distinct approaches: fusion with the double-stranded DNA-binding protein Sso7d and charge-altering mutations of a key residue designed to enhance binding to the DNA phosphate backbone. Two additional variants, Taq388 (Du et al., 2022) and TM-Taq (Lim et al., 2022), were engineered to enhance the specificity and mismatch sensitivity of Taq pol. Taq388 was developed by optimizing the interactions between the finger domain of Taq pol and the DNA template, whereas TM-Taq was developed by modifying the charge within the catalytic pocket relative to the primer 3′-terminal bases. Therefore, engineered Taq variants representing two distinct design strategies, strong binding affinity and high amplification specificity, were developed to assess their performance in TaqMan qPCR, thereby providing insights into enzyme performance improvement. Compared with Taq-wt, Taq388 and TM-Taq exhibit superior probe cleavage efficiency. This finding suggests that their engineered charge redistribution optimizes the kinetic interplay among 5′-3′ polymerase, 5′-3′ exonuclease, and strand displacement activities, thereby enhancing performance in TaqMan qPCR. By establishing a screening method that integrates these activities, the present study provides a basis for the development of high-performance TaqMan qPCR detection.

Additionally, this study demonstrates that although KlenTaq lacks exonuclease activity (Lorenz, 1974; Barnes, 1992; Lawyer et al., 1993), it can still perform probe detection by utilizing strand displacement. This property makes KlenTaq suitable for probe melting curve analysis and other applications. Thus, the findings presented herein expand the application scope of Taq pol in TaqMan qPCR and provide a new approach for identifying novel detection methods.

## 2 Results

### 2.1 Taq pol nuclease activity

To systematically investigate the cleavage activity of the Taq pol 5′-3′ exonuclease domain (N-terminal, 1–291 aa), multiple models were generated to compare the differences between its exonuclease and 5′-flap endonuclease activities. To strictly avoid potential interference from the polymerase activity (i.e., DNA extension), all experiments were conducted in the absence of dNTPs to more clearly examine the nuclease behavior and cleavage characteristics of Taq pol under various conditions.

**FIGURE 1:**
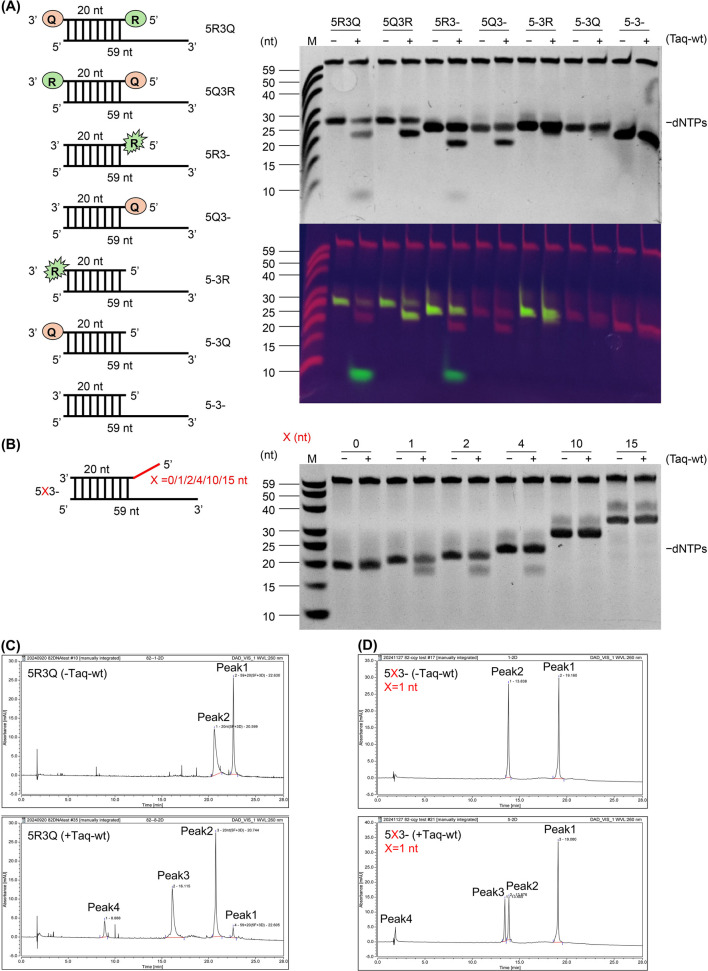
Nuclease activity assays of Taq pol. **(A)** Exonuclease activity was analyzed via a denaturing PAGE. Probes with different modifications (R = fluorescent group, Q = quencher, - = unmodified) were incubated with a common complementary template at 50 °C for 45 min, without dNTPs and with (+) or without (−) Taq-wt. Gel images and fluorescence signals were captured with a UV imaging system. **(B)** Endonuclease activity was analyzed via a denaturing PAGE. Probes with varying 5′-free arm lengths (X = length) were incubated with a common complementary template at 50 °C for 45 min without dNTPs and with (+) or without (−) Taq-wt, and gel patterns were analyzed. **(C,D)** Exonuclease activity **(C)** was assessed by ion chromatography using the 5R3Q probe and 5′-flap endonuclease activity **(D)** was assessed using the 5X3- (X = 1) probe. Probes were incubated with (+) or without (−) Taq-wt at 50 °C for 45 min without dNTPs. Peaks from 200 to 600 nm were analyzed; peak 1 = probe-template complex, peak 2 = intact probe, peak 3 = longer cleavage product, and peak 4 = shorter cleavage product.

To examine exonuclease activity, seven different test probes were allowed to anneal with a common complementary template and then incubated with or without Taq-wt at 50 °C for 45 min. The reaction products were then analyzed by denaturing polyacrylamide gel electrophoresis (PAGE). The results showed that the 5R3Q and 5R3-probes exhibited clear cleavage fragments, while the 5Q3R and 5Q3-probes were cleaved, but only one of the two expected fragments was observed on the PAGE. In contrast, 5-3R, 5-3Q, and 5-3- probes did not undergo any cleavage (Figure 1A). These findings indicate that Taq pol exhibits significant cleavage activity in probes with a 5′end fluorescent modification, but little activity was noted in probes without such a modification. Notably, when the reaction time was extended to three or 12 h, probes without a 5′-terminal fluorescent modification, as well as unmodified primers with no modifications at either end, were cleaved to varying degrees (Supplementary Figure S2A,B). Additionally, in the presence of dNTPs, the probe cleavage efficiency was significantly reduced when compared to an absence of dNTPs (Supplementary Figure S3A–D), suggesting that the presence of dNTPs strongly promotes polymerase activity over exonuclease activity.

To evaluate 5′-flap endonuclease activity, six different test probes with varying 5′-arm lengths and a free 5′-end were employed. These 5X3-probes were annealed to a common complementary template and then also incubated with or without Taq-wt at 50 °C for 45 min. Following denaturing PAGE analysis, the results showed clear cleavage bands for probes with X = 1, 2, and four nucleotides (nt), while no cleavage was detected for the other probes (Figure 1B). When the reaction time was extended to 3 h, the X = 10 nt probe also underwent cleavage, and the cleavage efficiency of the X = 1, 2, and 4 nt probes was further enhanced (Supplementary Figure S4A), indicating that extending the reaction time significantly improves 5′-flap endonuclease activity. Additionally, the addition of dNTPs significantly reduced the cleavage efficiency in samples X = 1, 2, and 4 nt (Supplementary Figure S4B), suggesting a bias towards polymerase activity. These results demonstrate that the endonuclease activity of Taq pol is affected by the 5′-end flap structure of the probe, and that a longer free 5′-arm results in weaker endonuclease activity.

Further analysis of the cleavage products using ion chromatography revealed that the 5R3Q probe, commonly used in TaqMan qPCR, produced two distinct product peaks (peak three and peak 4) after probe cleavage (Figure 1C). The 5R3-probe, which is less commonly used, showed similar peaks, and its ion chromatography data was provided in Supplementary Figure S5A. In contrast, the 5-3R probe showed no such peaks. Moreover, several 5X3-probes (X = 1, 2, 4 nt) also exhibited two distinct peaks relative to the control (Figure 1D; Supplementary Figure S5B). Additionally, single-stranded library sequencing of the reaction products showed that with the 5R3-probe, a 19-nt cleavage product accounted for approximately 84.81%, while the same-length cleavage product accounted for approximately 59.39% of the 5X3- (X = 4 nt) probe (Supplementary Figure S6A,B), When X = 4 nt, the 5′free arm is long enough to allow clear observation of cleavage by the 5′-flap endonuclease activity (e.g., generating 22-nt or 23-nt products). These results demonstrate that the cleavage site, whether mediated by the exonuclease or 5′-flap endonuclease activity of Taq pol, is most likely to occur between the first and second bases of the 5′end of the probe and the complementary region of the template.

In summary, both Taq pol exonuclease and 5′-flap endonuclease activities occur independent of primer extension and dNTPs. Exonuclease activity was found to exhibit a significantly higher cleavage efficiency in the presence of probes with a 5′end fluorescent modification compared to unmodified primers. Moreover, endonuclease activity appeared dependent on the probe having a 5′-end flap structure, but with an increased arm length decreasing the cleavage efficiency. Additionally, each enzymatic activity occurred only once during the qPCR reaction time, and primarily cleaved the phosphodiester bond between the first and second bases of the 5′-end pairing region of the probe.

### 2.2 The process of Taq pol-mediated probe cleavage

**FIGURE 2:**
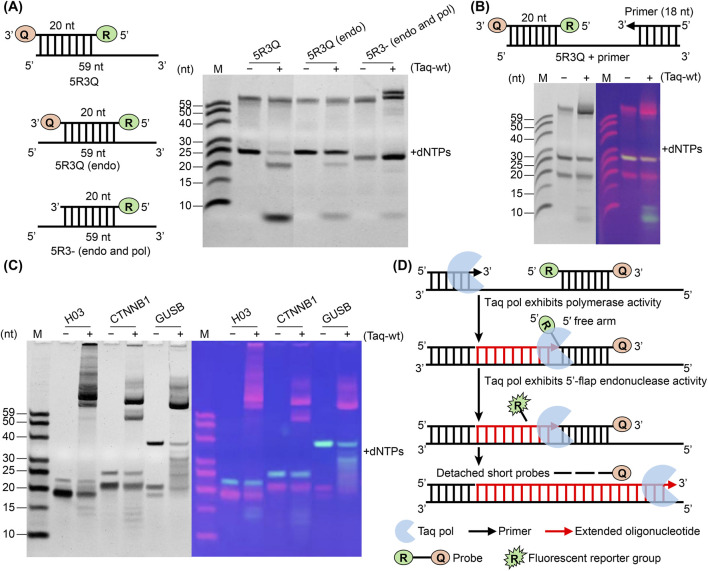
Mechanism of probe cleavage by Taq pol during primer extension. **(A)** Different probes were bound to different locations on a common complementary template to evaluate various combinations of nuclease and polymerase activities. Reactions were carried out with (+) or without (−) Taq-wt in the presence of dNTPs and were incubated at 50 °C for 45 min and analyzed via denaturing PAGE. **(B)** To evaluate polymerase extension, probe (5R3Q) and primer were combined (5R3Q + primer) with (+) or without (−) Taq-wt and incubated at 50 °C for 45 min in the presence of dNTPs. Reactions were analyzed by denaturing PAGE and visualized with a UV gel imaging system. **(C)** Probe cleavage analysis using three TaqMan qPCR systems (*H03*, *CTNNB1* and *GUSB*) was performed, followed by denaturing PAGE analysis with (+) or without (−) Taq-wt. The cycling conditions were 95 °C for 30 s, followed by 45 cycles of 95 °C for 30 s and 60 °C for 30 s. The results were analyzed with a UV gel imaging system. **(D)** Schematic of polymerase extension and probe cleavage by Taq pol.

To gain further insight into the mechanism of Taq pol in probe cleavage, three reaction models were constructed. The 5R3Q model, where the probe binds the 5′end of the template to promote exonuclease activity; the 5R3Q (endo) model features a probe that binds the middle region of the template, allowing both exonuclease and 5′-flap endonuclease activities due to the presence of the 5′-end flap structure; in contrast, the 5R3- (endo and pol) model uses a 3′-end unmodified probe that binds the middle region of the template, supporting exonuclease, 5′-flap endonuclease, and polymerase activities simultaneously (Figure 2A). After incubation at 50 °C for 45 min in the presence of dNTPs, the products were analyzed using a denaturing PAGE. The results showed that compared to the 5R3Q model, the 5R3Q (endo) model exhibited a significantly weaker probe cleavage band, while the 5R3- (endo and pol) model showed not only a weaker probe cleavage band, but also an additional band that was larger than 59 nt (Figure 2A). This larger band was likely due to an extension of the 3′end of the probe, resulting in an incompletely denatured double-stranded product. This suggests that when both the exonuclease and 5′-flap endonuclease activities are available, the exonuclease activity is significantly attenuated. When all three activities are available, polymerase activity is preferred while the others are suppressed. This finding is also consistent with the observation that the presence of dNTPs promotes polymerase activity over exonuclease activity.

To investigate probe cleavage during primer extension, a complementary primer was combined with the 5R3Q probe to create a 5R3Q + primer model. After incubation at 50 °C for 45 min in the presence of dNTPs, the products were analyzed as described above. The results showed that the probe was cleaved at multiple sites, with fluorescent fragments of various sizes observed (Figure 2B). Additionally, similar probe digestion results were observed when detecting three target genes, namely, *H03* (GenBank AC242242.3), *CTNNB1* (GenBank AB451392.1), and *GUSB* (GenBank AK300223.1) in the human 293 cell line using TaqMan qPCR (Figure 2C). This observation deviates from the above-mentioned result, which shows that in the absence of polymerase activity, exonuclease activity results in a single fluorescent fragment from the probe. These results suggest that, in addition to exonuclease activity, multiple other enzymatic activities are involved in the probe cleavage process.

Previous studies have reported that Taq pol can exhibit strand displacement activity, where the displaced strand region is subsequently cleaved by its 5′-3′ nuclease activity (Barnes et al., 2021). Based on the observed variation in cleavage products, we propose that when Taq pol encounters the probe, strand displacement occurs, generating a 5′free arm that subsequently provides the 5′-flap structural requirement for endonuclease activity. A portion of the probe is then cleaved at the nuclease active site and this cycle repeats until the probe becomes too short to remain on the template, after which Taq pol continues to extend the product until its completion (Figure 2D).

### 2.3 The probe affects the amplification performance of Taq pol

**FIGURE 3:**
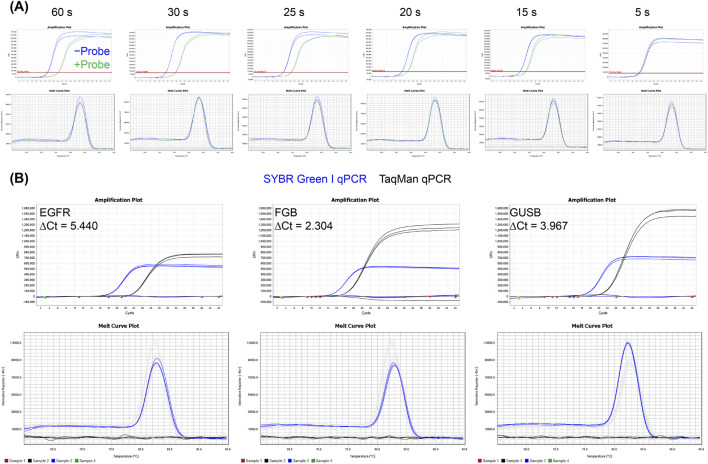
Effects of the probe on the amplification performance of Taq pol. **(A)** Effect of the presence of a probe on the melting curve when examining the *FGB* gene via TaqMan qPCR at different extension times. Ct values were compared between reactions with and without the probe to assess the probe’s impact. **(B)** Comparison of amplification performance between SYBR Green I and TaqMan qPCR. The three target genes (*EGFR*, *FGB* and *GUSB*) were detected using SYBR Green I and ROX channels on the same qPCR instrument. Ct value differences were used to evaluate probe effects on Taq pol performance. The melting curves were generated using SYBR Green I.

While polymerase extension and probe hydrolysis were shown to occur concomitantly, it was unclear if the presence of the probe may still inhibit the extension capability. To evaluate the impact of the probe on the amplification performance, the qPCR reaction targeting the human *FGB* gene (GenBank OR234395.1) was performed with or without probe using SYBR Green I. The results showed that the addition of the probe significantly reduced the amplification efficiency, with a more pronounced decrease observed at shorter extension times (Figure 3A; Supplementary Table S1).

The effect of a probe was further examined by comparing TaqMan (probe-based) and SYBR Green I (dye-based) qPCR systems. The three human target genes, *EGFR* (GenBank GU255993.1), *FGB* (GenBank OR234395.1), and *GUSB* (GenBank AK300223.1), were amplified and purified to serve as templates for qPCR, thereby ensuring the specificity of the amplification reaction. The result revealed a higher amplification plateau in TaqMan, but a significantly delayed Ct value (Figure 3B). While this delay might be attributed to the time required for polymerase-mediated probe displacement and cleavage, the differences between platforms arose from the distinct methods by which qPCR instruments captured fluorescence signals from dyes and probes, even when the amounts of amplified targets were comparable (Supplementary Figure S7).

Therefore, while the introduction of the probe enhances the specificity and amplification plateau of qPCR, it may also reduce the overall amplification efficiency of Taq pol, thereby prolonging the time required to reach the amplification plateau and ultimately affecting the detection sensitivity.

### 2.4 Screening of Taq variants with high cleavage efficiencies

In TaqMan qPCR, it is commonly believed that a Taq pol with strong 5′-3′ exonuclease activity is essential for efficient probe cleavage (Zhu et al., 2023). However, following systematic analysis of Taq pol nuclease activities during probe cleavage it was found that the 5′-3′ exonuclease activity was also closely related to polymerase activity, strand displacement activity, and 5′-flap endonuclease activity.

To evaluate these functions, the levels of polymerase extension and probe cleavage mediated by Taq variants were quantified. To enable a quantitative comparison between different Taq variants, the probe cleavage efficiency was defined as the ratio of probe cleavage to product yield and was utilized as an evaluation index. This method was applied to evaluate several previously reported Taq variants, including the high-processivity fusion protein S-Taq (Wang, 2004), the high-affinity variant Taq-E507K (Arezi et al., 2014), the high-specificity variant Taq388 (Du et al., 2022) and the mismatch recognition capability variant TM-Taq (Lim et al., 2022). These variants, including Taq-wt, were examined via qPCR with *EGFR*, *FGB* and *GUSB* genes examined. The results showed that for the Taq-wt, the average probe cleavage efficiency across the three genes, was 77.39%. S-Taq and Taq-E507K exhibited a slightly lower average efficiency of 76.87% and 72.16%, respectively, whereas Taq388 (91.96%) and TM-Taq (88.19%) showed a 1.2-fold higher average efficiency relative to the Taq-wt (Table 1).

**TABLE 1:** Probe cleavage efficiency of different Taq variants based on probe cleavage and product accumulation quantities.

Genes	Metrics	Taq-wt	S-Taq	Taq-E507K	Taq388	TM-Taq
EGFR	PC (pmol)	6.82	6.35	6.28	7.59	7.7
	PY (pmol)	8.96	8.47	8.95	8.84	8.85
	PC/PY (%)	76.12	74.97	70.17	85.86	87.01
FGB	PC (pmol)	7.06	7.18	6.45	8.34	8.29
	PY (pmol)	8.24	8.54	8.64	7.97	8.59
	PC/PY (%)	85.68	84.07	74.65	104.64	96.51
GUSB	PC (pmol)	5.53	5.29	5.41	6.19	6.46
	PY (pmol)	7.86	7.39	7.55	7.25	7.97
	PC/PY (%)	70.36	71.58	71.66	85.38	81.05

PC: probe cleavage amount; PY: amplified product quantity measured by Qubit; PC/PY: a ratio of probe cleavage efficiency, with higher values indicating a higher probe cleaved at the same product level.

**FIGURE 4:**
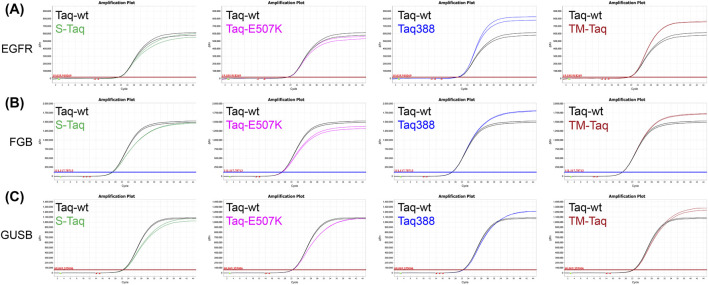
Comparison of amplification performance between Taq-wt and four variants in TaqMan qPCR. **(A–C)** *EGFR* **(A)**, *FGB* **(B)** and *GUSB* **(C)** were used to evaluate Taq variants (S-Taq, TaqE507K, Taq338, and TM-Taq) relative to Taq-wt based on amplification plots.

To further evaluate the amplification performance of these Taq variants, the amplification specificity was examined and all variants were found to have a comparable sensitivity to that of the Taq-wt (Supplementary Figure S8). Amplification plots were then constructed that compared each variant to the Taq-wt. Under conditions in which all Taq variants exhibited Ct values comparable to that of Taq-wt (ΔCt within ±0.5), S-Taq and Taq-E507K showed comparable or slightly lower plateaus when compared to the Taq-wt, whereas TM-Taq and Taq388 showed higher product numbers prior to reaching a plateau relative to the Taq-wt (Figure 4A–C; Supplementary Tables S2,S3). This suggests that TM-Taq and Taq388 demonstrate better coordination between probe cleavage and polymerase extension, effectively balancing the interaction between these two processes and thereby enhancing overall detection efficiency. These results also validate the feasibility of the established evaluation method, providing an effective strategy for screening and determining the applicability of various Taq variants.

### 2.5 Application of KlenTaq in TaqMan qPCR

**FIGURE 5:**
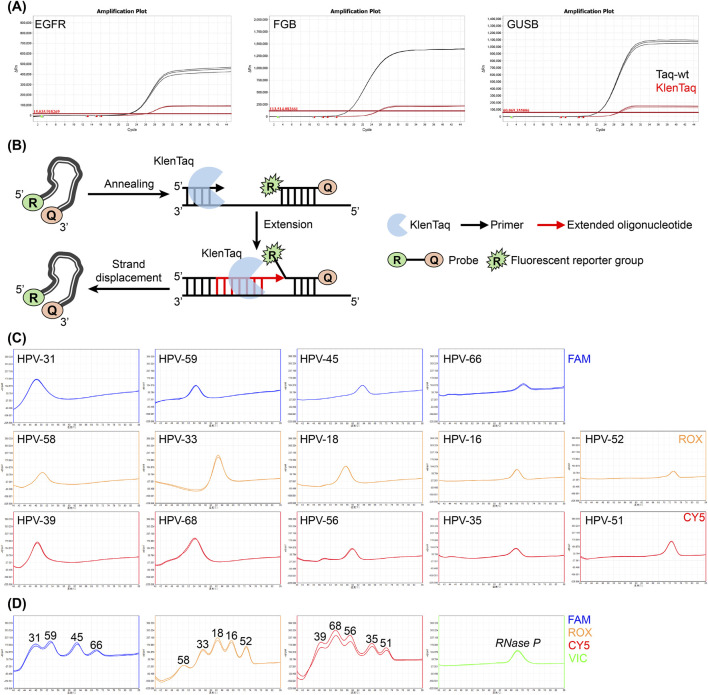
Application of KlenTaq in TaqMan qPCR. **(A)** Amplification plots comparing Taq-wt and KlenTaq in *EGFR*, *FGB* and *GUSB* genes. **(B)** Schematic of polymerase extension and probe displacement by KlenTaq during TaqMan qPCR. **(C)** Probe melting curve analysis used to detect 14 high-risk HPV subtypes (HPV-16, HPV-18, HPV-31, HPV-33, HPV-35, HPV-39, HPV-45, HPV-51, HPV-52, HPV-56, HPV-58, HPV-59, HPV-66 and HPV-68). Probes with three different reporters, FAM, ROX and Cy5, were combined with KlenTaq at a template concentration of 500 copies/µL to generate single-target curves. **(D)** Multi-target HPV detection in a single-tube reaction using a probe melting curve method. Probes were again combined with the same reporters at a template concentration of 500 copies/µL with *RNase P* (GenBank AC279469.1) used as an internal control and detected with VIC.

KlenTaq is a truncated variant of Taq pol, lacking 289 N-terminal amino acids that form the 5′-3′ exonuclease domain (Lawyer et al., 1993). Although previous studies have shown that KlenTaq exhibits higher fidelity and thermal stability, it is considered unsuitable for TaqMan qPCR due to its inability to perform probe cleavage, and its application has therefore remained limited (Barnes, 1992; Kermekchiev et al., 2009). Following amplification analysis, KlenTaq showed a delayed Ct and an amplification curve that was only slightly above the baseline (Figure 5A). To further investigate its underlying enzymatic mechanism, exonuclease and 5′-flap endonuclease activities were evaluated using the probes identified above, followed by denaturing PAGE, which showed an absence of probe cleavage in KlenTaq, consistent with its structure (Supplementary Figure S9A,B).

To further explore the mechanism by which KlenTaq interacts with the probe during extension, a primer was combined with the 5-3- probe, and a single-stranded library was constructed and sequenced. The results showed that in KlenTaq, approximately 97% of the reads corresponded to intact probe, which was significantly higher than the ∼4% that was observed in Taq-wt (Supplementary Figure S9C). To further evaluate KlenTaq, reactions were performed with 5R3Q + primer in the presence of dNTPs and denaturing PAGE analysis further confirmed that KlenTaq maintains an intact probe (Supplementary Figure S10). When considered collectively, these findings suggest that KlenTaq possesses a stronger strand displacement activity that effectively releases an intact probe so that extension can proceed (Figure 5B; Supplementary Figure S9C). During this displacement process, the conformation of the probe changes and a low level of fluorescence signal can be observed despite the quencher still being attached. Due to these characteristics, KlenTaq is particularly desirable for detection scenarios that require high probe integrity, such as probe melting curve analysis (Elenitoba-Johnson et al., 2001; Liao et al., 2013).

To evaluate the performance of KlenTaq in probe melting curve analysis, the human *MYC* gene (GenBank AH002907.2) was used as a target, and the detection efficiency between KlenTaq and Taq-wt were compared. The results showed that KlenTaq produces a higher derivative peak, indicating improved detection sensitivity (Supplementary Figure S11A). KlenTaq performance was further evaluated in disease screening, with 14 different subtypes of human papillomavirus (HPV) effectively detect using probes coupled with FAM, ROX or Cy5 reporters at a template concentration of 500 copies/μL (Figure 5C). Additionally, accurate subtyping was achieved in a multiplex, single-tube detection system utilizing the same reporters (Figure 5D). Even when the template concentration was reduced to 100 copies/μL, KlenTaq still maintained a good detection sensitivity and typing capability (Supplementary Figure S11B,C), indicating a strong application potential for probe melting curve detection.

## 3 Discussion

As noted in the Introduction (Holland et al., 1991; Lyamichev et al., 1993), the traditional model describes Taq pol as sequentially hydrolyzing the 5′-terminal nucleotides of the probe during extension; however, the precise impact on amplification efficiency remains unclear, and the relative contributions of strand displacement versus 5′-3′ exonuclease activity require further clarification (Barnes et al., 2021).

In the present work, the nuclease and polymerase activities of Taq pol were systematically examined to characterize the synergism required for probe cleavage and extension. Under conditions lacking a primer or dNTPs, Taq pol retained both 5′-3′ exonuclease (Holland et al., 1991; Lyamichev et al., 1993) and 5′-flap endonuclease activities (Lyamichev et al., 1993), indicating that its nuclease function operates autonomously (Lorenz, 1974), independent of the polymerase reaction (Figures 1A,B). Additionally, Taq pol exhibited weak probe cleavage activity in the presence of unmodified probes or probes lacking a 5′-fluorescent group (Figures 1A,B), suggesting that fluorescent modifications may enhance probe recognition and cleavage efficiency. However, although the exploration of diverse 5′-fluorescent modifications offers potential to optimize probe detection sensitivity and cleavage efficiency, critical gaps remain regarding the specific contributions of fluorophore identity, size, linker flexibility, and position to the conformational dynamics of the enzyme-probe complex (Huang and Li, 2009).

In Taq pol, the processes of polymerase extension and probe cleavage are not entirely independent, with polymerase activity typically prioritized. Upon reaching the probe during polymerase extension, the probe is displaced and cleavage occurs via 5′-flap endonuclease activity, with this process repeated until the probe is completely hydrolyzed and while polymerase extension proceeds (Figure 2D). Obtaining the ratio of the probe cleavage amount to product accumulation amount provided a theoretical basis for screening cleavage efficiency and can be effectively used as an evaluation criterion. Furthermore, analyzing the amplification efficiencies of Taq variants identified Taq388 and TM-Taq as having improved efficiencies over Taq-wt (Figures 4A–C; Supplementary Table S3). Consequently, TM-Taq and Taq388 exhibit a more harmonized catalytic profile, in which the precise coordination of cleavage and extension amplifies overall detection efficiency. However, although the performance of these optimized variants under standard cycling conditions for single target gene detection has been validated, several critical limitations hinder their immediate translation into real diagnostic scenarios, particularly with respect to ultra-low viral loads, point-of-care rapid detection, and complex sample matrices (Bustin, 2010). Crucially, the underlying structure-function relationships of these superior variants remain unclear. Without high-resolution structural data or dynamic modeling, it remains difficult to fully characterize how mutations at specific residues stabilize the conformational ensembles required for efficient cleavage (Yabukarski, 2025). Addressing this knowledge gap will be critical for further optimizing these enzymes for high-precision diagnostic applications. Furthermore, the optimal balance between probe cleavage kinetics and polymerase extension rates during simultaneous reactions remains to be determined (Schwartz and Quake, 2009).

The potential applications and limitations of KlenTaq (lacking the 5′-3′ exonuclease domain) were evaluated. Although KlenTaq does not possess exonuclease or 5′-flap endonuclease activity (Lorenz, 1974; Barnes, 1992; Lawyer et al., 1993), it exhibits a strong strand displacement capability (Supplementary Figure S7A–C). Unlike traditional TaqMan assays, in which probes are irreversibly consumed and background noise commonly arises from non-specific cleavage (Han et al., 2022), KlenTaq preserves probe integrity by physically displacing probes rather than cleaving them. This mechanism reduces baseline noise and maintains a high concentration of intact probe-template complexes, resulting in sharper and more intense melting peaks, which improves the clarity of target discrimination and facilitates unambiguous interpretation of complex multiplex results. Furthermore, when combining KlenTaq with various dyes and employing a melting curve analysis (Elenitoba-Johnson et al., 2001; Liao et al., 2013), KlenTaq was able to distinguish 14 single-target HPV subtypes and showed good sensitivity when employed in a single-tube multiplex assay (Figures 5C,D; Supplementary Figure S9B,C).

In Taq pol, the processes of polymerase extension and probe cleavage are not entirely independent; polymerase activity is typically prioritized, yet a precise synergistic interplay governs efficient probe hydrolysis. The present study bridges mechanistic insights with practical optimization to identify superior Taq variants with balanced cleavage-extension kinetics and validates KlenTaq as a robust alternative that preserves probe integrity for high-resolution melting curve analysis. Collectively, these findings provide a framework for engineering next-generation qPCR systems, offering a valuable strategy for developing efficient and specific molecular diagnostic methods with broad application prospects in complex pathogen detection and multiplex analysis.

## Data Availability

The original contributions presented in the study are included in the article/Supplementary Material; further inquiries can be directed to the corresponding author.

## Supplementary material

SUPPLEMENTARY FIGURE S1 SDS-PAGE verification of Taq-wt and Taq variants. The molecular weights of the detected proteins were Taq-wt (94 kDa), Taq-E507K (94 kDa), Taq388 (94 kDa), TM-Taq (94 kDa), KlenTaq (62.4 kDa) and S-Taq (102 kDa), respectively.

SUPPLEMENTARY FIGURE S2 Effect of reaction time on Taq pol exonuclease activity. **(A,B)** Examination of exonuclease activity using seven probes with (+) or without (−) Taq-wt and without dNTPs that were incubated for 3 h **(A)** and 12 h **(B)** at 50 °**(C)** and analyzed by denaturing PAGE.

SUPPLEMENTARY FIGURE S3 Effect of dNTPs on Taq pol exonuclease activity. **(A)** Comparison of exonuclease activity in the presence or absence of dNTPs. The 5R3Q probe was incubated at 50 °C for 45 min, fluorescence was monitored and an amplification plot was constructed. **(B**–**D)** Detection of seven probes in the presence of dNTPs, with (+) or without (−) Taq-wt, that were incubated at 50 °C for 45 min **(B)**, 3 h **(C)**, and 12 h **(D)**. Exonuclease activity was determined based on denaturing PAGE analysis.

SUPPLEMENTARY FIGURE S4 Effect of prolonged reaction time on the 5′-flap endonuclease activity of Taq pol. **(A,B)** Six probes were utilized to evaluate 5′-flap endonuclease activity both without dNTPs **(A)** and with dNTPs **(B)** at 50 °C for 3 h, with (+) or without (−) Taq-wt, and analyzed by denaturing PAGE.

SUPPLEMENTARY FIGURE S5 Influence of the fluorescent modification position and the 5′-arm length on Taq pol exonuclease activity. **(A,B)** Exonuclease activity **(A)** was assessed by ion chromatography using the 5R3-and 5-3R probes and 5′-flap endonuclease activity **(B)** was assessed using the 5X3- (X = 2 and 4) probes. Probes were incubated with (+) or without (−) Taq-wt at 50 °C for 45 min without dNTPs. Peaks from 200 to 600 nm were analyzed; peak 1 = probe-template complex, peak 2 = probe, peak 3 = longer cleavage product, peak 4 = shorter cleavage product, and peak 5 = cleavage product + template complex.

SUPPLEMENTARY FIGURE S6 Determination of probe cleavage sites. **(A,B)** Single-stranded library sequencing was used to determine the cleavage sites during exonuclease activity **(A)** using the 5R3-probe and during 5′-flap endonuclease activity **(B)** using the 5X3- (X = 4) probe. The products were sequenced and the proportion of each cleavage product was calculated based on the total reads.

SUPPLEMENTARY FIGURE S7 Comparison of platform performance between SYBR Green I and TaqMan qPCR. Three different target genes (*EGFR*, *FGB* and *GUSB*) were selected, and each was simultaneously detected in a single reaction tube via SYBR Green I and the ROX channel. Under conditions where amplicon specificity and yield were identical, platform differences in distinct fluorescence detection methods were observed. Melting curves were generated using SYBR Green I.

SUPPLEMENTARY FIGURE S8 Product specificity of Taq-wt and Taq variants. The products from Taq-wt and four variants were obtained, followed by qPCR analysis of the *EGFR*, *FGB* and *GUSB* genes. Products were resolved by agarose gel electrophoresis, with a target band of approximately 100 bp detected for each.

SUPPLEMENTARY FIGURE S9 The nuclease and strand-displacement activities of KlenTaq. **(A,B)** Exonuclease activity **(A)** was analyzed using the 5R3Q probe and 5′-flap endonuclease activity **(B)** was examined using the 5X3- (X = 4) probe. Reactions were incubated at 50 °C for 45 min, 3 h and 12 h under dNTPs-free conditions. Products were evaluated via denaturing PAGE and visualized using a UV gel imaging system. Taq-wt was used as control. **(C)** Strand-displacement activity was evaluated using single-stranded library sequencing. The 5-3- + primer model was incubated at 50 °C for 45 min under dNTPs-containing conditions, followed by sequencing. The proportion of intact probe reads relative to the total probe reads was quantified.

SUPPLEMENTARY FIGURE S10 The influence of KlenTaq on the probe during primer extension. The 5R3Q + primer model was incubated with or without Taq-wt or with KlenTaq at 50 °C for 45 min under dNTPs-containing conditions. Products were analyzed by denaturing PAGE, and band patterns and fluorescence signals were visualized using a UV gel imaging system.

Supplementary Figure S11 Performance of KlenTaq in probe melting curve analysis. **(A)** Comparison of Taq-wt and KlenTaq in detecting the human *MYC* gene. **(B)** Detection of 14 high-risk HPV subtypes (HPV-16, HPV-18, HPV-31, HPV-33, HPV-35, HPV-39, HPV-45, HPV-51, HPV-52, HPV-56, HPV-58, HPV-59, HPV-66 and HPV-68) using probe melting curves. Probes with three different reporters, FAM, ROX and Cy5, were combined with KlenTaq at a template concentration of 100 copies/µL. **(C)** Multi-target HPV detection in a single-tube reaction using a probe melting curve method. Probes were again combined with the same reporters at a template concentration of 100 copies/µL and *RNase P* was used as an internal control and detected using VIC.

## References

[B1] Arezi B. McKinney N. Hansen C. Cayouette M. Fox J. Chen K. (2014). Compartmentalized self-replication under fast PCR cycling conditions yields Taq DNA polymerase mutants with increased DNA-binding affinity and blood resistance. Front. Microbiol. 5, 408. 10.3389/fmicb.2014.00408 25177317 PMC4132270

[B2] Barnes W. M. (1992). The fidelity of Taq polymerase catalyzing PCR is improved by an N-terminal deletion. Gene 112 (1), 29–35. 10.1016/0378-1119(92)90299-5 1551596

[B3] Barnes W. M. Zhang Z. Kermekchiev M. B. (2021). A single amino acid change to Taq DNA polymerase enables faster PCR, reverse transcription and strand-displacement. Front. Bioeng. Biotechnol. 8, 553474. 10.3389/fbioe.2020.553474 33520948 PMC7841393

[B4] Brock T. D. Freeze H. (1969). *Thermus aquaticus* gen. n. and sp. n., a Nonsporulating extreme thermophile. J. Bacteriol. 98 (1), 289–297. 10.1128/JB.98.1.289-297.1969 5781580 PMC249935

[B5] Bustin S. A. (2010). Developments in real-time PCR research and molecular diagnostics. Expert Rev. Mol. diagn. 10 (6), 713–715. 10.1586/erm.10.65 20843195

[B6] Chen C.-Y. (2014). DNA polymerases drive DNA sequencing-by-synthesis technologies: both past and present. Front. Microbiol. 5, 305. 10.3389/fmicb.2014.00305 25009536 PMC4068291

[B7] Chien A. Edgar D. B. Trela J. M. (1976). Deoxyribonucleic acid polymerase from the extreme thermophile Thermus aquaticus. J. Bacteriol. 127 (3), 1550–1557. 10.1128/jb.127.3.1550-1557.1976 8432 PMC232952

[B8] Du P. Li B. Liu X. Yang L. Ren N. Li Y. (2022). Enhanced Taq variant enables efficient genome editing testing and mutation detection. CRISPR J. 5 (1), 131–145. 10.1089/crispr.2021.0105 35076264

[B9] Eischeid A. C. (2011). SYTO dyes and EvaGreen outperform SYBR green in real-time PCR. BMC Res. Notes 4 (1), 263. 10.1186/1756-0500-4-263 21798028 PMC3162529

[B10] Elenitoba-Johnson K. S. J. Bohling S. D. Wittwer C. T. King T. C. (2001). Multiplex PCR by multicolor fluorimetry and fluorescence melting curve analysis. Nat. Med. 7 (2), 249–253. 10.1038/84708 11175859

[B11] Fouchier R. A. M. Bestebroer T. M. Herfst S. Van Der Kemp L. Rimmelzwaan G. F. Osterhaus A. D. M. E. (2000). Detection of influenza A viruses from different species by PCR amplification of conserved sequences in the matrix gene. J. Clin. Microbiol. 38 (11), 4096–4101. 10.1128/JCM.38.11.4096-4101.2000 11060074 PMC87547

[B12] Han Y. Liu L. Liu Q. Wang S. (2022). Optimization and performance evaluation of double-stranded probe in real-time PCR. Anal. Biochem. 650, 114711. 10.1016/j.ab.2022.114711 35561816

[B13] Holland P. M. Abramson R. D. Watson R. Gelfand D. H. (1991). Detection of specific polymerase chain reaction product by utilizing the 5′-3′ exonuclease activity of Thermus aquaticus DNA polymerase. Proc. Natl. Acad. Sci. 88 (16), 7276–7280. 10.1073/pnas.88.16.7276 1871133 PMC52277

[B14] Huang Q. Li Q. (2009). Characterization of the 5′ to 3′ nuclease activity of thermus aquaticus DNA polymerase on fluorogenic double-stranded probes. Mol. Cell. Probes 23 (3–4), 188–194. 10.1016/j.mcp.2009.04.002 19376219

[B15] Huang W.-F. Zhang Y. Mehmood S. Wang Z. Hou C. Li Z. (2021). Updating sacbrood virus quantification PCR method using a TaqMan-MGB probe. Vet. Sci. 8 (4), 63. 10.3390/vetsci8040063 33924550 PMC8070565

[B16] Innis M. A. Myambo K. B. Gelfand D. H. Brow M. A. (1988). DNA sequencing with Thermus aquaticus DNA polymerase and direct sequencing of polymerase chain reaction-amplified DNA. Proc. Natl. Acad. Sci. 85 (24), 9436–9440. 10.1073/pnas.85.24.9436 3200828 PMC282767

[B17] Kermekchiev M. B. Kirilova L. I. Vail E. E. Barnes W. M. (2009). Mutants of Taq DNA polymerase resistant to PCR inhibitors allow DNA amplification from whole blood and crude soil samples. Nucleic Acids Res. 37 (5), e40. 10.1093/nar/gkn1055 19208643 PMC2655666

[B18] Khodakov D. Wang C. Zhang D. Y. (2016). Diagnostics based on nucleic acid sequence variant profiling: PCR, hybridization, and NGS approaches. Adv. Drug Deliv. Rev. 105 (Pt A), 3–19. 10.1016/j.addr.2016.04.005 27089811

[B19] Kim Y. Eom S. H. Wang J. Lee D.-S. Suh S. W. Steitz T. A. (1995). Crystal structure of Thermus aquaticus DNA polymerase. Nature 376 (6541), 612–616. 10.1038/376612a0 7637814

[B20] Lawyer F. C. Stoffel S. Saiki R. K. Chang S. Y. Landre P. A. Abramson R. D. (1993). High-level expression, purification, and enzymatic characterization of full-length Thermus aquaticus DNA polymerase and a truncated form deficient in 5’ to 3’ exonuclease activity. Genome Res. 2 (4), 275–287. 10.1101/gr.2.4.275 8324500

[B21] Li B. Liu J. Huang Q. (2023). A digital PCR method based on highly specific Taq for detecting gene editing and mutations. Int. J. Mol. Sci. 24 (17), 13405. 10.3390/ijms241713405 37686219 PMC10488114

[B22] Li Z. Yang X. Yang B. Yang J. Tao C. Zhang D. (2025). A comprehensive review of methodological and technological advancement in PCR during the last 15 years. Biotechnol. Adv. 85, 108719. 10.1016/j.biotechadv.2025.108719 41022185

[B23] Liao Y. Zhou Y. Guo Q. Xie X. Luo E. Li J. (2013). Simultaneous detection, genotyping, and quantification of human papillomaviruses by multicolor real-time PCR and melting curve analysis. J. Clin. Microbiol. 51 (2), 429–435. 10.1128/JCM.02115-12 23175255 PMC3553893

[B24] Lim Y. Park I.-H. Lee H.-H. Baek K. Lee B.-C. Cho G. (2022). Modified Taq DNA polymerase for allele-specific ultra-sensitive detection of genetic variants. J. Mol. Diagn. 24 (11), 1128–1142. 10.1016/j.jmoldx.2022.08.002 36058471 PMC9746316

[B25] Lorenz V. (1974). Vesiculated tuffs and associated features. Sedimentology 21 (2), 273–291. 10.1111/j.1365-3091.1974.tb02059.x

[B26] Lyamichev V. Brow M. A. D. Dahlberg J. E. (1993). Structure-specific endonucleolytic cleavage of nucleic acids by eubacterial DNA polymerases. Science 260 (5109), 778–783. 10.1126/science.7683443 7683443

[B27] Morrison T. B. Weis J. J. Wittwer C. T. (1998). Quantification of low-copy transcripts by continuous SYBR green I monitoring during amplification. BioTechniques 24 (5), 960–962. 10.1006/abio.2000.4753 9631186

[B28] Mullis K. B. Faloona F. A. (1987). Specific synthesis of DNA *in vitro via* a polymerase-catalyzed chain reaction. Methods Enzymol. 155, 335–350. 10.1016/0076-6879(87)55023-6 3431465

[B29] Mullis K. Faloona F. Scharf S. Saiki R. Horn G. Erlich H. (1986). Specific enzymatic amplification of DNA *in vitro:* the polymerase chain reaction. Cold Spring Harb. Symp. Quant. Biol. 51, 263–273. 10.1101/SQB.1986.051.01.032 3472723

[B30] Saiki R. K. Gelfand D. H. Stoffel S. Scharf S. J. Higuchi R. Horn G. T. (1988). Primer-directed enzymatic amplification of DNA with a thermostable DNA polymerase. Science 239 (4839), 487–491. 10.1126/science.2448875 2448875

[B31] Schmittgen T. D. Zakrajsek B. A. Mills A. G. Gorn V. Singer M. J. Reed M. W. (2000). Quantitative reverse transcription-polymerase chain reaction to study mRNA decay: comparison of endpoint and real-time methods. Anal. Biochem. 285 (2), 194–204. 11017702 10.1006/abio.2000.4753

[B32] Schwartz J. J. Quake S. R. (2009). Single molecule measurement of the “speed limit” of DNA polymerase. Proc. Natl. Acad. Sci. 106 (48), 20294–20299. 10.1073/pnas.0907404106 19906998 PMC2787106

[B33] Shahrajabian M. H. Sun W. (2024). The significance and importance of dPCR, qPCR, and SYBR green PCR kit in the detection of numerous diseases. Curr. Pharm. Des. 30 (3), 169–179. 10.2174/0113816128276560231218090436 38243947

[B34] Tozaki T. Ohnuma A. Iwai S. Kikuchi M. Ishige T. Kakoi H. (2021). Robustness of digital PCR and real-time PCR in transgene detection for gene-doping control. Anal. Chem. 93 (18), 7133–7139. 10.1021/acs.analchem.1c01173 33913315

[B35] Wang Y. Prosen D. E. Mei L. Sullivan J. C. Finney M. Vander Horn P. B. (2004). A novel strategy to engineer DNA polymerases for enhanced processivity and improved performance *in vitro* . Nucleic Acids Res. 32 (3), 1197–1207. 10.1093/nar/gkh271 14973201 PMC373405

[B36] Wilhelm J. Pingoud A. (2003). Real‐time polymerase chain reaction. ChemBioChem 4 (11), 1120–1128. 10.1002/cbic.200300662 14613102

[B37] Yabukarski F. (2025). Ensemble-function relationships: from qualitative to quantitative relationships between protein structure and function. J. Struct. Biol. 217 (1), 108152. 10.1016/j.jsb.2024.108152 39577782

[B38] Zhu Z. Sun D. Quimby A. (2023). Taq DNA polymerase mutants for probe qPCR. Available online at: https://patents.google.com/patent/US11649441B2/en (Accessed December 16, 2025).

