# Supplementary figures and images for: Mechanism and application of Taq DNA polymerase in TaqMan qPCR
Source: Front Bioeng Biotechnol. 2026 May 20;14:1773703. doi: 10.3389/fbioe.2026.1773703 (PMC13230210; doi:10.3389/fbioe.2026.1773703)

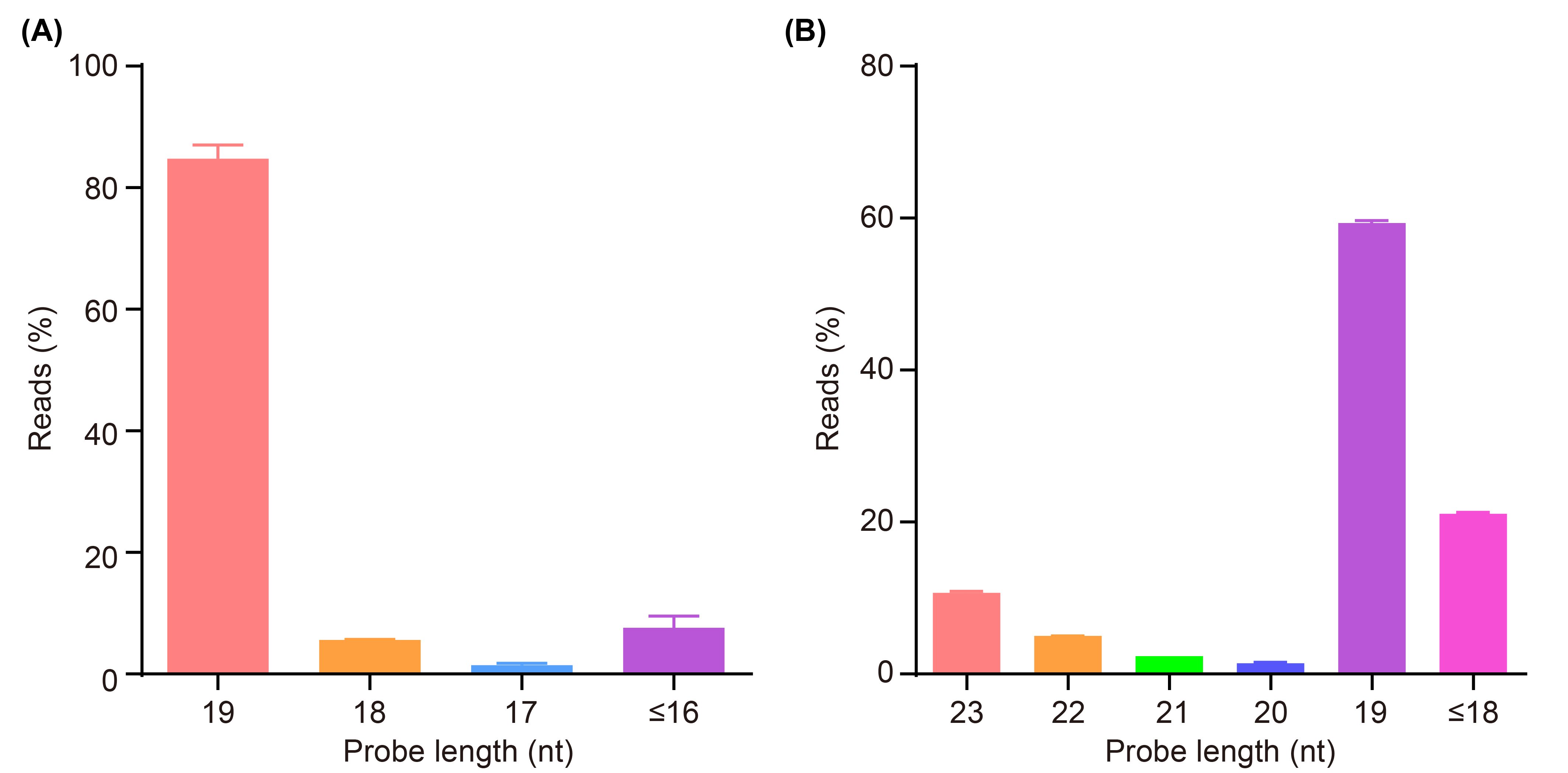

Supplement: Supplementary file 1 [file Image6.tif]

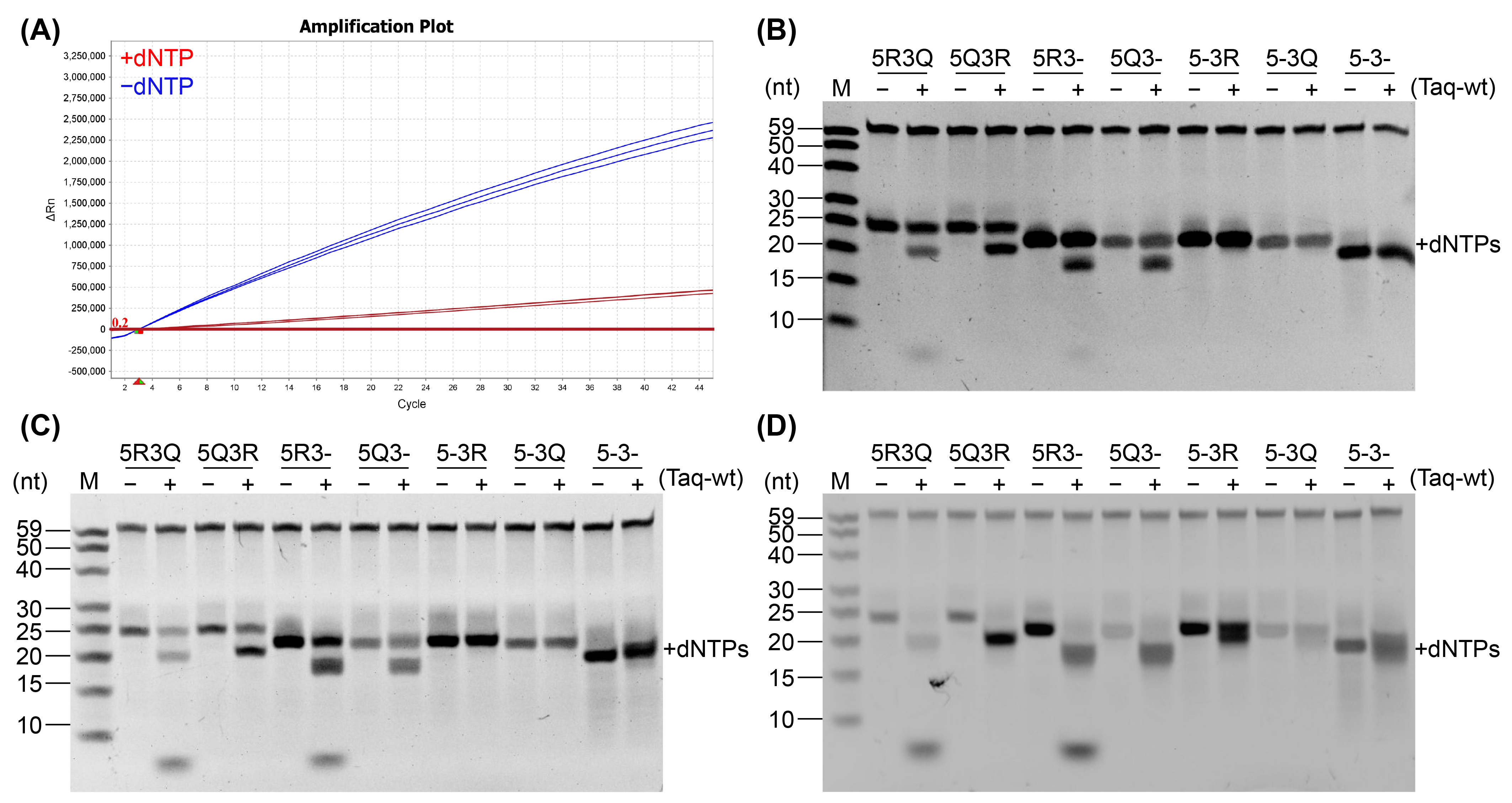

Supplement: Supplementary file 3 [file Image3.tif]

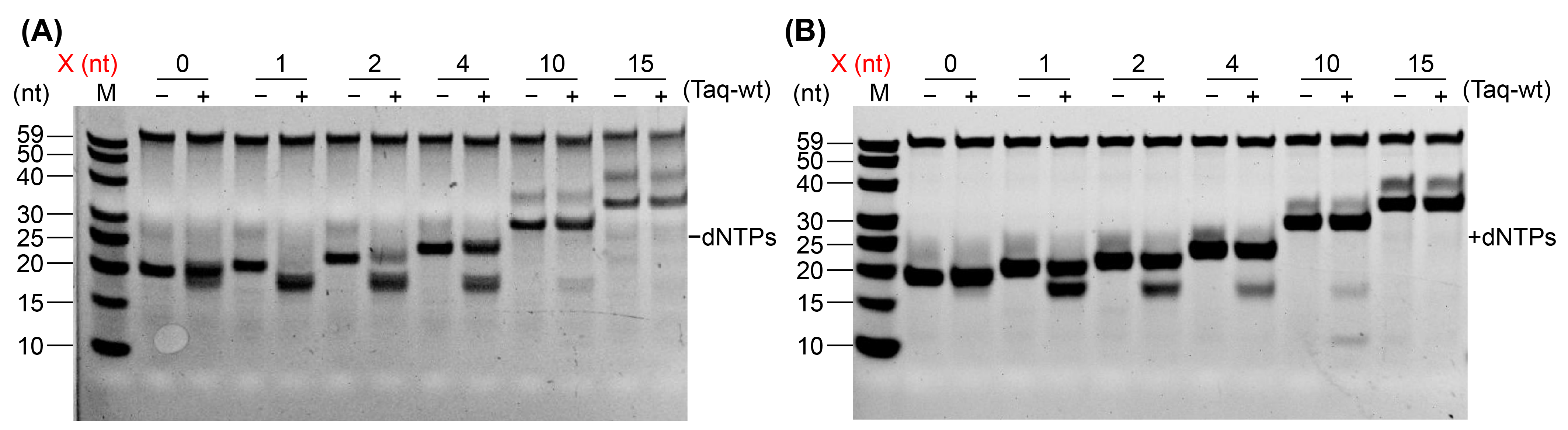

Supplement: Supplementary file 4 [file Image4.tif]

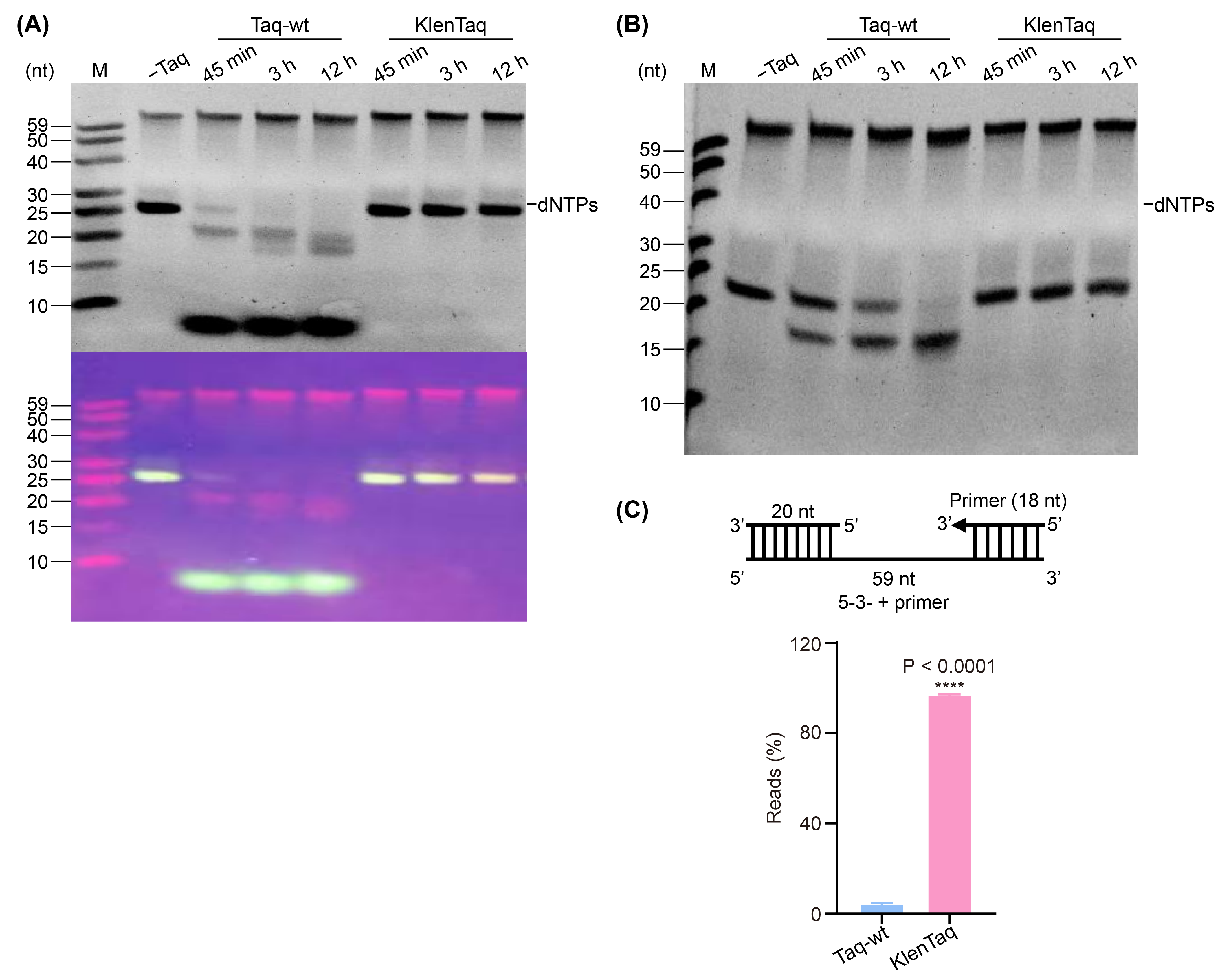

Supplement: Supplementary file 5 [file Image9.tif]

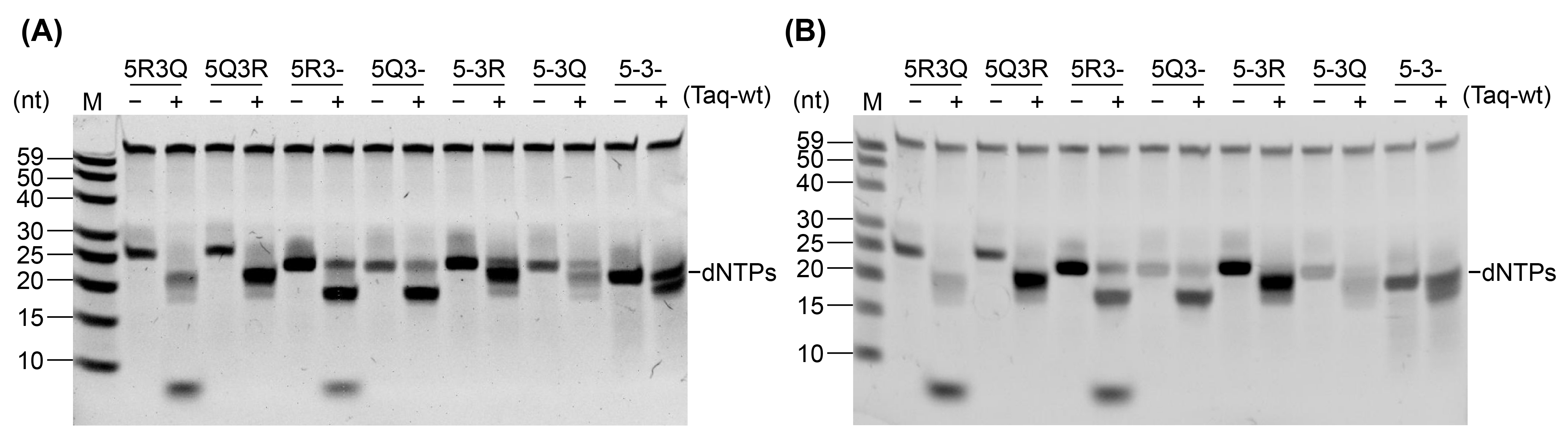

Supplement: Supplementary file 6 [file Image2.tif]

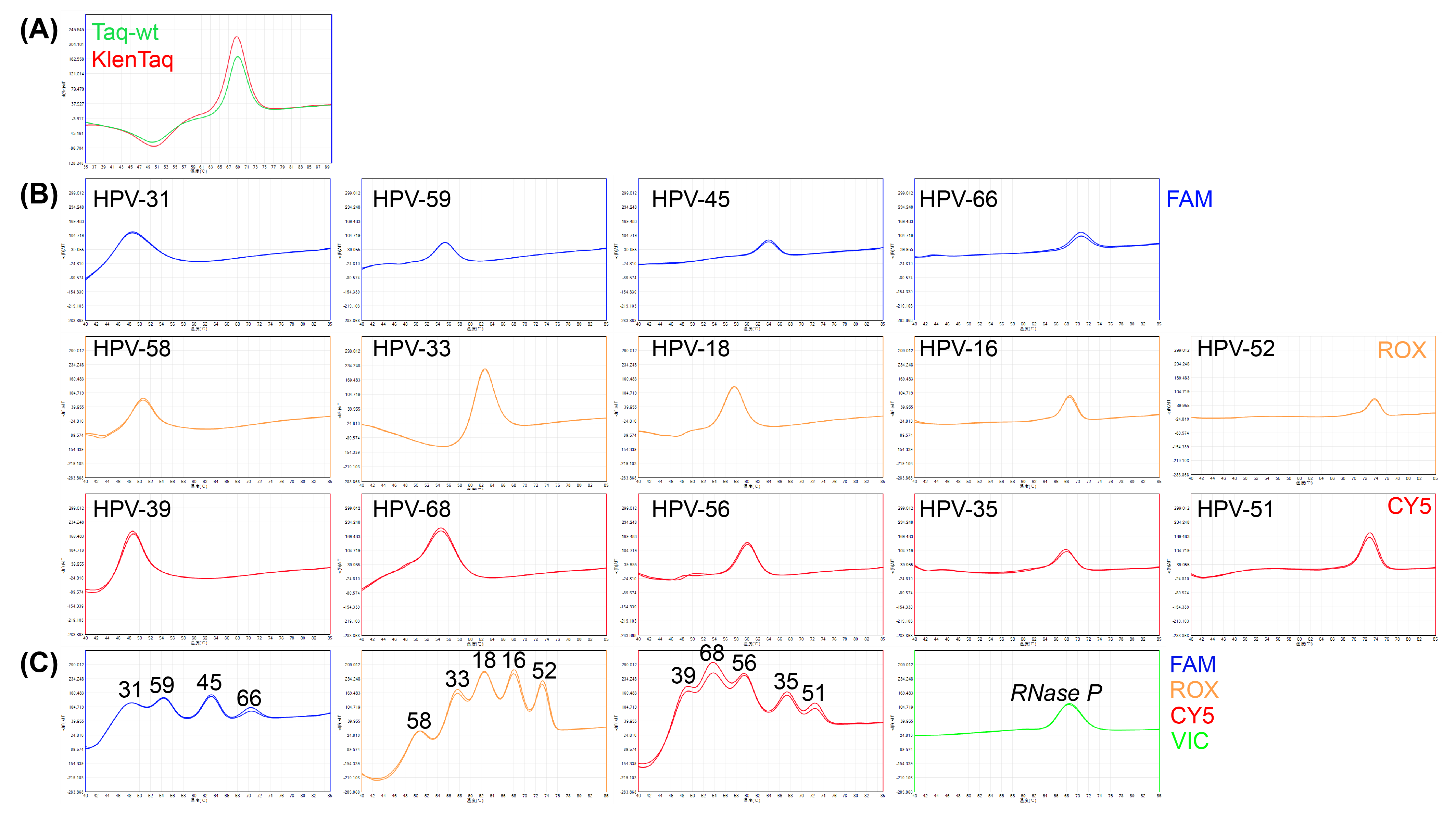

Supplement: Supplementary file 7 [file Image11.tif]

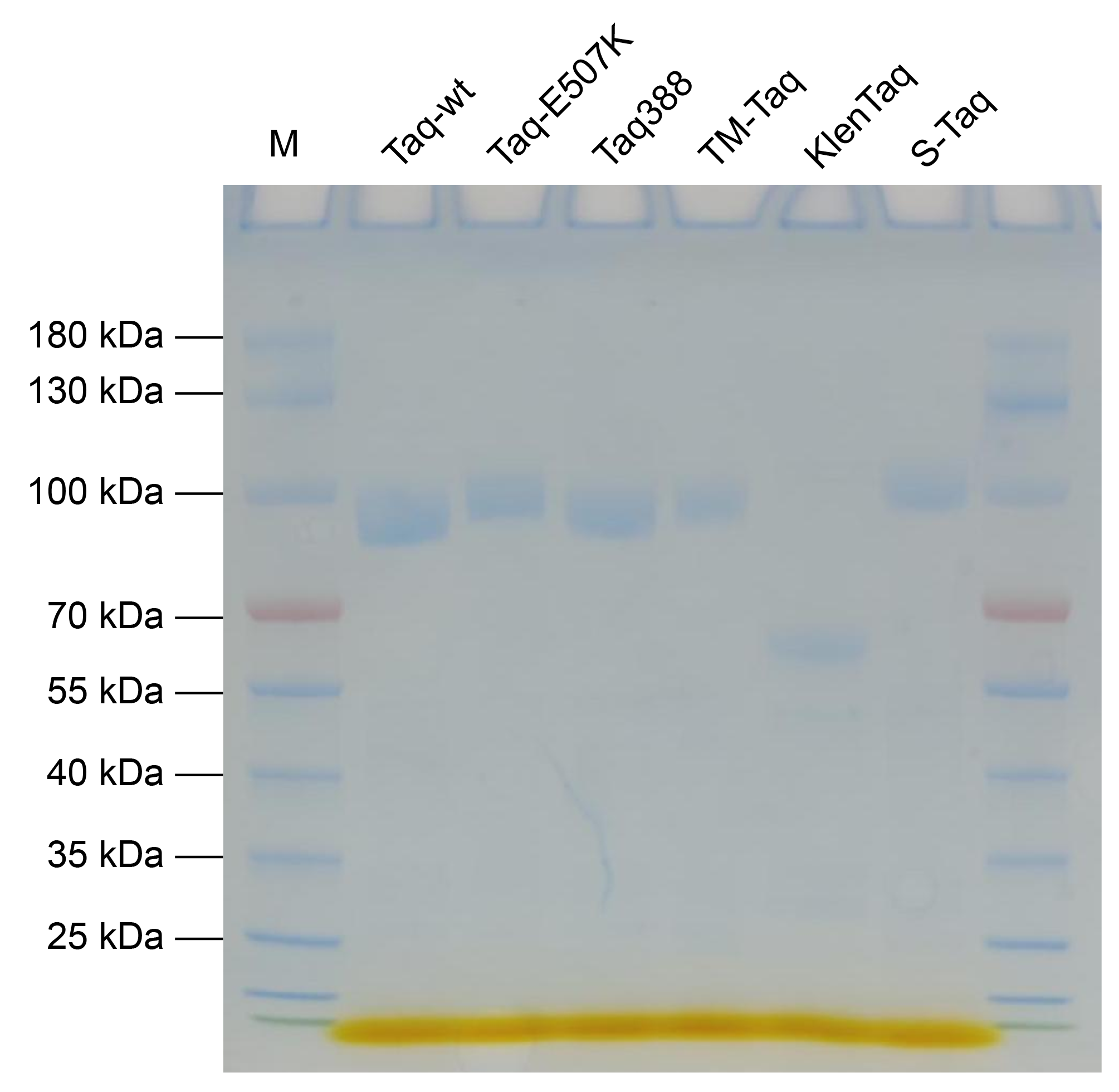

Supplement: Supplementary file 8 [file Image1.tif]

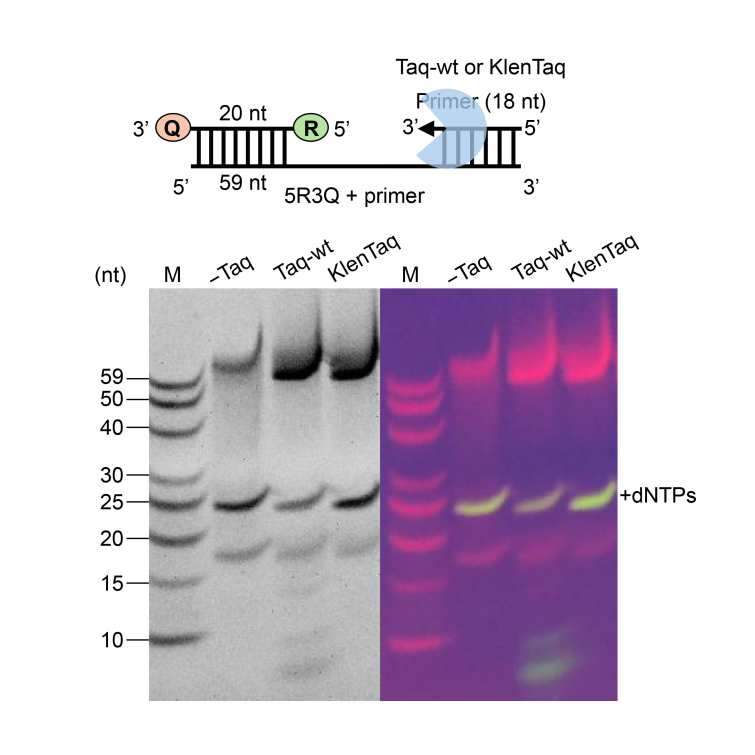

Supplement: Supplementary file 9 [file Image10.tif]

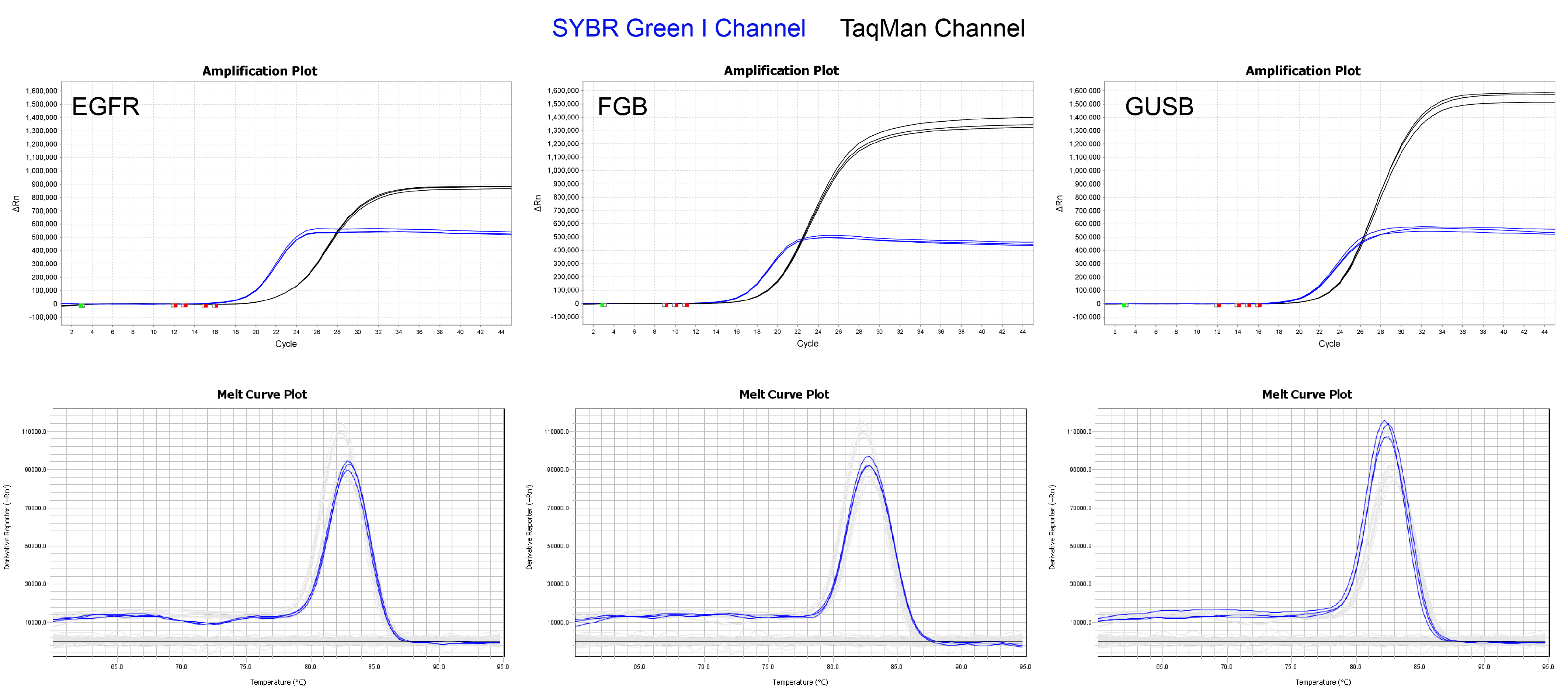

Supplement: Supplementary file 10 [file Image7.tif]

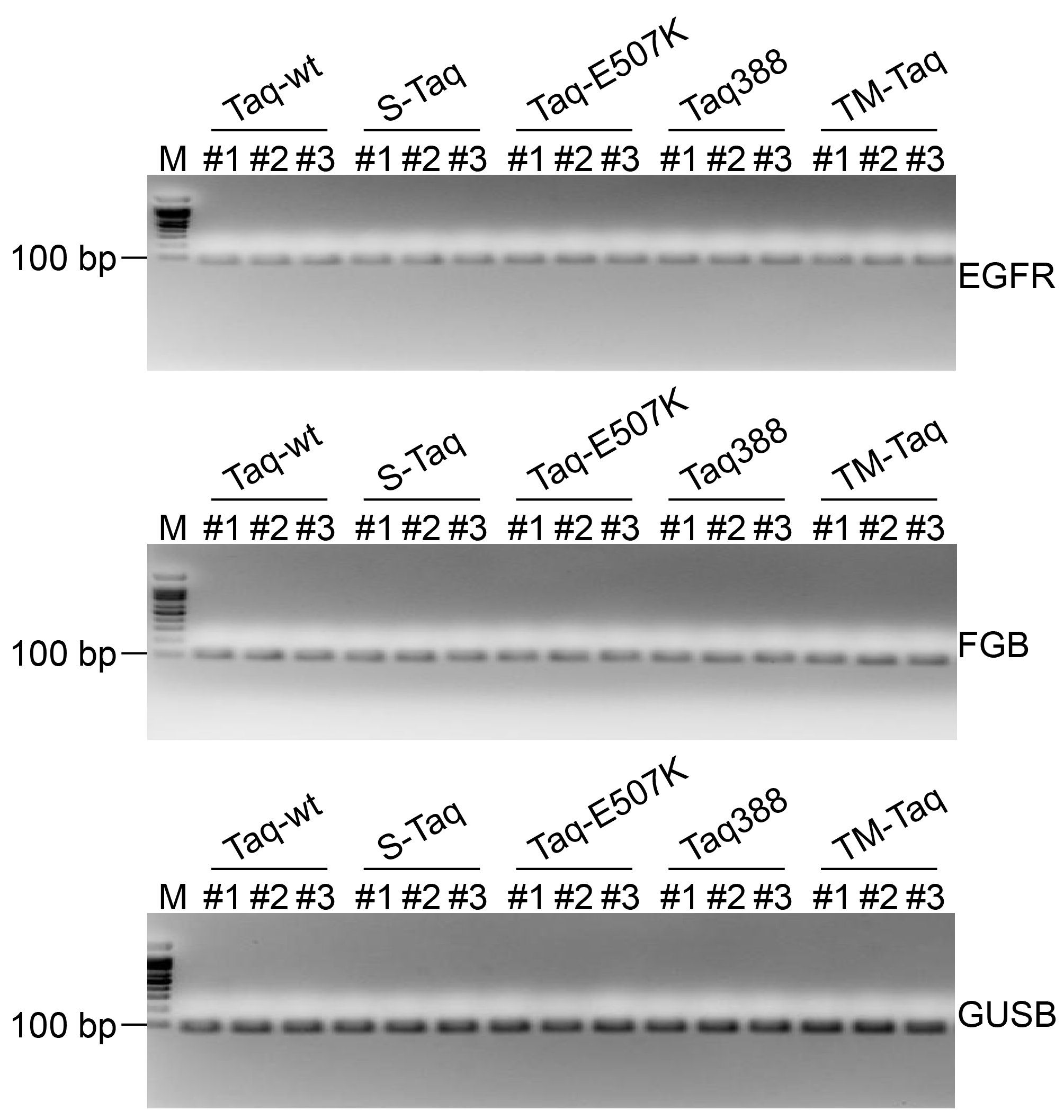

Supplement: Supplementary file 12 [file Image8.tif]

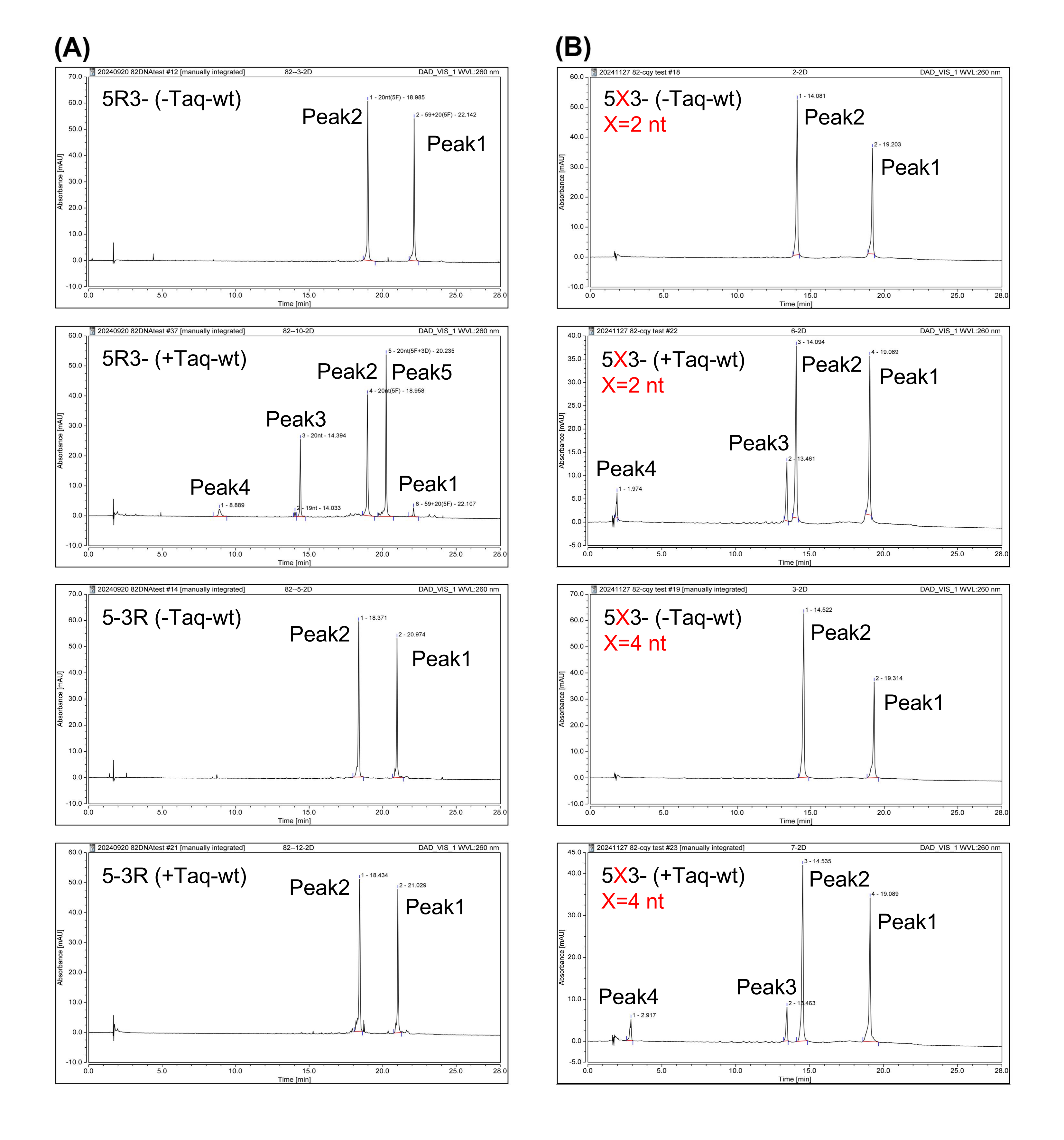

Supplement: Supplementary file 13 [file Image5.tif]
