# Supplementary material for: Mechanism and application of Taq DNA polymerase in TaqMan qPCR
Source: Front Bioeng Biotechnol. 2026 May 20;14:1773703. doi: 10.3389/fbioe.2026.1773703 (PMC13230210; doi:10.3389/fbioe.2026.1773703)
Supplement: Supplementary file 2 [file Supplementaryfile1.docx]

Supplementary Material

# Methods

## Plasmid construction and protein preparation

To construct the vector for enzyme expression and purification, specific primers and the ClonExpress Ultra One Step Cloning Kit V2 (Catalog No. C216, Vazyme Biotech, Nanjing, China) were used. The Taq-wt gene (GenBank BAA06775) was PCR-amplified using Phanta Max Super-Fidelity DNA Polymerase (Catalog No. P505, Vazyme Biotech, Nanjing, China) with primers Taq-F and Taq-R. The pET-28a-SUMO plasmid (Catalog No. P0028, Zhifang Biotech, Shanghai, China) was PCR-amplified using primers pET-28a-Taq-F and pET-28a-Taq-R. The two fragments were then assembled into a complete plasmid (pET-28a-Taq-wt) via homologous recombination. Sso7d (GenBank ACX90648.1) was PCR-amplified using primers S-Taq-F and S-Taq-R. The pET-28a-Taq-wt plasmid was PCR-amplified using primers pET-28a-Taq-F and pET-28a-Taq-R, and the two fragments were subsequently assembled into a complete plasmid (pET-28a-S-Taq). The pET-28a-Taq-E507k plasmid was obtained by PCR amplification using primers Taq-E507K-F and Taq-E507K-R via homologous recombination. For Taq388, three fragments were PCR-amplified using primers Taq388-F1/R1, Taq388-F2/R2, and Taq388-F3/R3, and the fragments were then assembled into a complete plasmid (pET-28a-Taq388). Similarly, for TM-Taq, three fragments were PCR-amplified using primers TM-Taq-F1/R1, TM-Taq-F2/R2, and TM-Taq-F3/R3, and the fragments were assembled into a complete plasmid (pET-28a-TM-Taq). The pET-28a-KlenTaq (Δ289) plasmid was obtained by PCR amplification using primers KlenTaq-F and KlenTaq-R via homologous recombination. All the above-mentioned primer sequences are listed in Supplementary Table S4, and the amplification and recombination procedures were performed according to the C216 kit manual. All plasmids were confirmed by sequencing at Sangon Biotech.

Proteins were expressed in *E. coli* BL21 (DE3) cells (Catalog No. C504, Vazyme Biotech, Nanjing, China) transformed with recombinant pET-28a-SUMO plasmids encoding His-tagged Taq-wt, Taq variants, and KlenTaq. Transformation was performed according to the instructions in the C504 manual. Transformed cells were cultured in LB medium supplemented with kanamycin at 37 °C until reaching an OD600 of 0.6–0.8. The cells were then induced with 1 mM IPTG at 16 °C overnight. After centrifugation at 12,000 rpm for 10 min, the bacterial pellet was resuspended in PBS and lysed by sonication. The crude lysate was heat-denatured at 72 °C for 30 min, and the supernatant was purified by Ni^2+^ affinity chromatography (Catalog No. HP201, Vazyme Biotech, Nanjing, China). The procedure was performed according to the instructions in the HP201 manual, and the His-tag was subsequently removed using SUMO protease (Catalog No. JE1004, Vazyme Biotech, Nanjing, China). The purified proteins were dialyzed and verified by SDS-PAGE for purity and specificity and then used for activity assays and functional studies (Supplementary Figure S1). At 74 °C, the amount of enzyme required to consume 10 nmol of dNTPs within 30 min was defined as 1 active unit (U).

## Test models assays

A target nucleic acid reaction system was constructed using oligonucleotides purchased from Shanghai Sangon Biotech Co., Ltd. (Shanghai, China), which were purified by polyacrylamide gel electrophoresis (PAGE). Oligonucleotides were mixed in defined ratios (probe:template = 4:1) and diluted in annealing buffer (50 mM Tris-HCl, pH 8.0; 200 mM KCl; 1.5 mM MgCl_2_). After mixing, the reaction mixture was denatured at 95 °C for 5 min, followed by a slow cooling process from 95 °C to 25 °C at 0.1 °C/s to allow efficient annealing and formation of stable double-stranded or secondary structures.

All probe model reactions were conducted in a MiniAmp Thermal Cycler (Thermo Fisher Scientific, Waltham, MA, USA) using 2.5 U of Taq-wt or Taq variants. Reaction components, including buffer and dNTPs, were provided with Champagne Taq DNA Polymerase (Catalog No. P122, Vazyme Biotech, Nanjing, China), and reaction mixtures were prepared according to the manufacturer’s instructions. All reactions were performed in a total volume of 20 µL. The reaction mixture contained 10× Champagne Taq Buffer (Mg^2+^ plus), 20 pmol template, and 80 pmol probe, with or without dNTPs and Taq polymerase as required. The samples were incubated at 50 °C for 45 min, 3 h, and 12 h.

## TaqMan qPCR and SYBR Green I qPCR assays

qPCR experiments were performed using a QuantStudio 3 Real-Time PCR System (Thermo Fisher Scientific, Waltham, MA, USA) with Champagne Taq DNA Polymerase kits (Catalog No. P122, Vazyme Biotech, Nanjing, China). Reactions were conducted in a 20 μL total volume containing 2.5 U of Taq-wt or Taq variants, dNTPs, and 10 μM primers. Thermal cycling conditions were identical for both assays: an initial denaturation at 95 °C for 30 s, followed by 45 cycles of denaturation at 95 °C for 30 s and annealing/extension at 60 °C for 30 s, with fluorescence detection at each cycle.

For TaqMan qPCR, a 10 μM probe was included in the primer mixture, and fluorescence was monitored specifically for the TaqMan probe during the extension phase. For SYBR Green I qPCR, following amplification, reaction specificity was assessed by generating a melting curve, which involved heating the samples to 95 °C for 15 s, cooling to 60 °C for 1 min, and then continuously monitoring fluorescence while ramping the temperature from 60 °C to 95 °C at a rate of 0.15 °C/s. Specificity was confirmed based on the melting curve profiles, including peak shape and melting temperature values.

## Denaturing polyacrylamide gel electrophoresis (PAGE)

A 20% denaturing polyacrylamide gel was prepared by mixing Acryl/Bis 30% Solution (29:1) (Catalog No. B546017, Sangon Biotech, Shanghai, China) and 20× TBE buffer (Catalog No. G103, Vazyme Biotech, Nanjing, China), followed by boiling. After cooling to room temperature, 10% APS (Catalog No. A100486, Sangon Biotech, Shanghai, China) and TEMED (Catalog No. A610508, Sangon Biotech, Shanghai, China) were added, and the mixture was rapidly mixed and poured into a gel cassette. The gel was allowed to polymerize at room temperature. Electrophoresis was carried out on a vertical system (Bio-Rad Mini-Protean Tetra) at 120 V for 20 min as a pre-run, followed by loading of samples mixed with 2× TBE-urea loading buffer (Catalog No. C506046, Sangon Biotech, Shanghai, China) and electrophoresis for 90–120 min at 120 V until the bromophenol blue dye had migrated approximately 3/4 of the gel. The gel was then removed and visualized under UV light (GenoSens2100, Clinx), and images were captured directly using the GenoSens capture system and a camera under the ultraviolet lamp.

## Ion chromatography

To confirm the anionic nature of the phosphate backbone present in the target nucleic acids, ion-exchange chromatography (IEX) was used to separate components based on charge differences. The analysis was performed on a Thermo Vanquish UHPLC system (Thermo Scientific, Waltham, MA, USA) using a gradient elution protocol with a 25 μL injection volume. Analytes were resolved on a Proteomix SAX-NP column (Sepax Technologies, Newark, DE, USA), which selectively interacts with negatively charged oligonucleotides. Separation was monitored using a UV-Vis detector (Dionex ICS-500, Thermo Scientific) across 200–600 nm. Data acquisition and spectral analysis were performed using the Thermo Scientific™ Dionex™ Chromeleon™ Chromatography Data System. The resulting profiles were identified by comparing retention times, peak shapes, and UV spectra with standard solutions of known oligonucleotide sequences and concentrations.

## Single-stranded library construction and sequencing

Single-stranded DNA libraries were constructed using the VAHTS ssDNA Library Preparation Kit (Catalog No. ND620, Vazyme Biotech, Nanjing, China) according to the manufacturer’s protocols. An Equalbit 1× dsDNA Assay Kit (Catalog No. EQ121, Vazyme Biotech, Nanjing, China) was used for DNA quantification to ensure quality. Qualified libraries were sequenced on an Illumina NovaSeq 6000 platform (Modi et al., 2021). Raw data were processed using Illumina’s Savant software for initial quality control, followed by analysis with FastQC and trimming with Trimmomatic to remove low-quality reads and adapters. The cleaned reads were aligned to the reference sequences, and the lengths and cleavage positions of each read were determined. The proportion of reads corresponding to each cleavage product relative to the total reads was then calculated.

## Assessment of probe cleavage efficiency

The TaqMan qPCR reaction was carried out using the QuantStudio 3 Real-Time PCR System (Thermo Fisher Scientific, Waltham, MA, USA), with the reaction system and conditions as described above. Upon completion of the TaqMan qPCR reaction, the reaction products were collected and analyzed to determine both the total product yield and the probe cleavage efficiency for each sample.

The dsDNA qPCR products were quantified using an Equalbit 1× dsDNA HS Assay Kit (Catalog No. EQ121, Vazyme Biotech, Nanjing, China) according to the manufacturer’s protocols. After completion of qPCR, a portion of the reaction product was mixed with Equalbit dye and quantified using a Qubit 3.0 Fluorometer (Thermo Fisher Scientific, Waltham, MA, USA). This value was then multiplied by the total reaction volume to determine the total product yield. To confirm product specificity, a portion of the reaction was analyzed on an agarose gel to verify amplicon size and exclude non-specific amplification or primer dimer formation.

Probe cleavage was assessed by fluorescence detection using a QuantStudio 3 Real-Time PCR System (Thermo Fisher Scientific, Waltham, MA, USA). A standard curve was generated by reacting different probe concentrations with nuclease (Catalog No. EN401, Vazyme Biotech, Nanjing, China) under identical conditions and recording fluorescence intensities. For experimental samples, fluorescence values were compared with the standard curve to quantify probe cleavage. Cleavage efficiency was calculated as the ratio of cleaved probe to total product.

## Probe melting curve analysis

The qPCR reaction was performed using a BioSmart U+ Melt Pro Multiplex DNA Polymerase Kit (Catalog No. PM301, Vazyme Biotech, Nanjing, China) according to the manufacturer’s protocol. The reaction mixture included 5 U of KlenTaq or Taq-wt, 10 μM primers and probes for 14 HPV subtypes and internal controls, and 5 µL of HPV gDNA control (Batch No. 270026–201101, Jinglang Biotech, Shenzhen, China). Amplification was carried out on a SLAN-6 thermocycler (Hongshi, Shanghai, China) with the following program: 37 °C for 2 min, 95 °C for 1 min, followed by 55 cycles at 95 °C for 15 s, 50 °C for 20 s, and 68 °C for 20 s. Following amplification, a melting curve was automatically generated by the instrument under the following conditions: heating to 95 °C for 1 min and then cooling to 35 °C for 1 min, with the melting curve recorded from 40 °C to 85 °C at a rate of 0.05 °C/s. Fluorescence was continuously monitored, and specificity and typing efficiency were assessed based on the melting curve profiles, including peak shape and melting temperature (Tm) values.

# Supplementary Figures and Tables

## Supplementary Figures

**Supplementary Figure 1.** SDS-PAGE verification of Taq-wt and Taq variants. The molecular weights of the detected proteins were Taq-wt (94 kDa), Taq-E507K (94 kDa), Taq388 (94 kDa), TM-Taq (94 kDa), KlenTaq (62.4 kDa) and S-Taq (102 kDa), respectively.

**Supplementary Figure 2.** Effect of reaction time on Taq pol exonuclease activity. **(A, B)** Examination of exonuclease activity using seven probes with (+) or without (–) Taq-wt and without dNTPs that were incubated for 3 h (**A**) and 12 h (**B**) at 50 °C and analyzed by denaturing PAGE.

**Supplementary Figure 3.** Effect of dNTPs on Taq pol exonuclease activity. **(A)** Comparison of exonuclease activity in the presence or absence of dNTPs. The 5R3Q probe was incubated at 50 °C for 45 min, fluorescence was monitored and an amplification plot was constructed. **(B**-**D)** Detection of seven probes in the presence of dNTPs, with (+) or without (–) Taq-wt, that were incubated at 50 °C for 45 min **(B)**, 3 h **(C)**, and 12 h **(D)**. Exonuclease activity was determined based on denaturing PAGE analysis.

**Supplementary Figure 4.** Effect of prolonged reaction time on the 5′-flap endonuclease activity of Taq pol. **(A, B)** Six probes were utilized to evaluate 5′-flap endonuclease activity both without dNTPs **(A)** and with dNTPs **(B)** at 50 °C for 3 h, with (+) or without (–) Taq-wt, and analyzed by denaturing PAGE.

**Supplementary Figure 5.** Influence of the fluorescent modification position and the 5′-arm length on Taq pol exonuclease activity. **(A, B)** Exonuclease activity **(A)** was assessed by ion chromatography using the 5R3- and 5-3R probes and 5′-flap endonuclease activity **(B)** was assessed using the 5X3- (X = 2 and 4) probes. Probes were incubated with (+) or without (–) Taq-wt at 50 °C for 45 min without dNTPs. Peaks from 200–600 nm were analyzed; peak 1 = probe-template complex, peak 2 = probe, peak 3 = longer cleavage product, peak 4 = shorter cleavage product, and peak 5 = cleavage product + template complex.

**Supplementary Figure 6.** Determination of probe cleavage sites. **(A, B)** Single-stranded library sequencing was used to determine the cleavage sites during exonuclease activity **(A)** using the 5R3- probe and during 5′-flap endonuclease activity **(B)** using the 5X3- (X = 4) probe. The products were sequenced and the proportion of each cleavage product was calculated based on the total reads.

**Supplementary Figure 7.** Comparison of platform performance between SYBR Green I and TaqMan qPCR. Three different target genes (*EGFR*, *FGB* and *GUSB*) were selected, and each was simultaneously detected in a single reaction tube via SYBR Green I and the ROX channel. Under conditions where amplicon specificity and yield were identical, platform differences in distinct fluorescence detection methods were observed. Melting curves were generated using SYBR Green I.

**Supplementary Figure 8.** Product specificity of Taq-wt and Taq variants. The products from Taq-wt and four variants were obtained, followed by qPCR analysis of the *EGFR*, *FGB* and *GUSB* genes. Products were resolved by agarose gel electrophoresis, with a target band of approximately 100 bp detected for each.

**Supplementary Figure 9.** The nuclease and strand-displacement activities of KlenTaq. **(A, B)** Exonuclease activity **(A)** was analyzed using the 5R3Q probe and 5′-flap endonuclease activity **(B)** was examined using the 5X3- (X = 4) probe. Reactions were incubated at 50 °C for 45 min, 3 h and 12 h under dNTPs-free conditions. Products were evaluated via denaturing PAGE and visualized using a UV gel imaging system. Taq-wt was used as control. **(C)** Strand-displacement activity was evaluated using single-stranded library sequencing. The 5-3-+primer model was incubated at 50 °C for 45 min under dNTPs-containing conditions, followed by sequencing. The proportion of intact probe reads relative to the total probe reads was quantified.

**Supplementary Figure 10.** The influence of KlenTaq on the probe during primer extension. The 5R3Q+primer model was incubated with or without Taq-wt or with KlenTaq at 50 °C for 45 min under dNTPs-containing conditions. Products were analyzed by denaturing PAGE, and band patterns and fluorescence signals were visualized using a UV gel imaging system.

**Supplementary Figure 11.** Performance of KlenTaq in probe melting curve analysis. **(A)** Comparison of Taq-wt and KlenTaq in detecting the human *MYC* gene. **(B)** Detection of 14 high-risk HPV subtypes (HPV-16, HPV-18, HPV-31, HPV-33, HPV-35, HPV-39, HPV-45, HPV-51, HPV-52, HPV-56, HPV-58, HPV-59, HPV-66 and HPV-68) using probe melting curves. Probes with three different reporters, FAM, ROX and Cy5, were combined with KlenTaq at a template concentration of 100 copies/µL. **(C)** Multi-target HPV detection in a single-tube reaction using a probe melting curve method. Probes were again combined with the same reporters at a template concentration of 100 copies/µL and *RNase P* was used as an internal control and detected using VIC.

## Supplementary Tables

**Supplementary Table 1.** Effect of the probe on Taq pol Ct values at different extension times.

|  | Extension time (s) | | | | | |
| --- | --- | --- | --- | --- | --- | --- |
|  | 60 | 30 | 25 | 20 | 15 | 5 |
| Ct (–probe) | 15.058 | 14.785 | 14.857 | 14.799 | 15.144 | 14.896 |
| Ct (+probe) | 15.319 | 17.370 | 17.619 | 18.208 | 19.455 | 19.896 |
| ∆Ct | 0.261 | 2.586 | 2.762 | 3.408 | 4.311 | 5.000 |

**Supplementary Table 2.** Comparison of amplification sensitivities among different Taq variants in TaqMan qPCR.

|  | S-Taq | Taq-E507K | Taq388 | TM-Taq |
| --- | --- | --- | --- | --- |
| EGFR  ∆Ct (mut–wt) | 0.47 | 0.23 | −0.07 | 0.24 |
| FGB  ∆Ct (mut–wt) | 0.09 | 0.13 | −0.11 | −0.08 |
| GUSB  ∆Ct (mut–wt) | 0.17 | 0.13 | 0.20 | 0.27 |

**Supplementary Table 3.** Comparison of amplification plateau differences among different Taq variants in TaqMan qPCR.

|  | S-Taq | Taq-E507K | Taq388 | TM-Taq |
| --- | --- | --- | --- | --- |
| EGFR  ∆Rn (mut–wt) / Rn (wt) | −9% | −15% | 28% | 24% |
| FGB  ∆Rn (mut–wt) / Rn (wt) | 3% | −10% | 21% | 18% |
| GUSB  ∆Rn (mut–wt) / Rn (wt) | 0% | −3% | 13% | 12% |

**Supplementary Table 4.** The nucleic acid sequences used in the study.

# References

Modi, A., Vai, S., Caramelli, D., and Lari, M. (2021). The illumina sequencing protocol and the NovaSeq 6000 system. *Methods Mol. Biol.* 2242, 15–42. doi: 10.1007/978-1-0716-1099-2_2
